# Recent thymic emigrants preferentially undergo memory inflation after persistent infection

**DOI:** 10.1371/journal.ppat.1013382

**Published:** 2025-07-28

**Authors:** Zachary T. Hilt, Arnold Reynaldi, Megan Steinhilber, Shide Zhang, Samantha P. Wesnak, Norah L. Smith, Miles P. Davenport, Brian D. Rudd

**Affiliations:** 1 Department of Microbiology and Immunology, Cornell University, Ithaca, New York, United States of America; 2 Kirby Institute, University of New South Wales, Sydney, New South Wales, Australia; The University of Alabama at Birmingham Department of Pediatrics, UNITED STATES OF AMERICA

## Abstract

Cytomegalovirus (CMV) leads to a unique phenomenon known as ‘memory inflation,’ where antigen-specific memory CD8 + T cells continue to accumulate in the peripheral tissues during the latent stage of infection. However, it is still not clear how the inflating pool of memory CD8 + T cells is generated and maintained. In this study, we used murine cytomegalovirus (MCMV) as a model of persistent infection and fate-mapping mice to determine the dynamics of CD8 + T cell recruitment into the memory pool. We found that neonatal exposure to CMV leads to an expansion of newly produced CD8 + T cells called recent thymic emigrants, or RTEs, which are maintained in the long-lived memory compartment. In contrast, CD8 + T cells produced after the acute phase of infection contribute minimally to memory inflation. We also observed notable phenotypic differences between the RTEs and mature CD8 + T cells that were recruited into the memory inflation response. Whereas the RTEs present at the time of infection gave rise to more effector memory cells, the mature CD8 + T cells were biased towards becoming central memory cells. Importantly, the preferential recruitment of RTEs into the effector memory pool also occurs during adult exposure to CMV. Collectively, these data demonstrate that persistent infection expands the RTE population, and timing of infection dictates whether neonatal or adult RTEs are ‘locked in’ to the memory pool.

## Author summary

Following infection with CMV, CD8 + T cells accumulate in the blood and peripheral organs over time, a feature termed ‘memory inflation’. However, it is not clear whether memory inflation is due to the continuous recruitment of cells that are produced during the latent stage of infection or expansion of CD8 + T cells that were present at the time of infection. To address this question, we used a fate-mapping mouse model and examined the recruitment of CD8 + T cells that were produced during different stages of infection. Surprisingly, we discovered that CD8 + T cells exported from the thymus just prior to infection are preferentially recruited and maintained in the memory pool. In contrast, CD8 + T cells produced during the latent stage contribute minimally to the inflating pool and exhibit a less differentiated phenotype. These results provide a new conceptual framework for understanding how the memory pool is generated and maintained after persistent viral infection.

## Introduction

Cytomegalovirus (CMV) is one of the most ubiquitous persistent viral infections in humans worldwide [1]. Depending on the geographical location, 12–56% of people become seropositive for CMV within the first year of life [2]. As a result, infants and children serve as a major source of infection in the community [3]. Transmission of human CMV (HCMV) most often occurs through contact with bodily fluids (urine, oral secretions, blood) of individuals actively shedding virus, with increased risk of exposure to HCMV in settings such as daycares [4]. Infection with CMV leads to a phenomenon known as ‘memory inflation,’ where antigen-specific CD8 + T cells accrue over time [5–10]. These inflating CD8 + T cells have an effector memory phenotype characterized by low expression of CCR7, CD62L, CD27, CD122, and CD127 and high expression of KLRG1 and CX3CR1 [6,11]. Despite being terminally differentiated, CMV-specific cells do not express markers of exhaustion (e.g., PD1, Lag3) and remain functional throughout the course of infection [6,12]. However, what is problematic is that CMV-specific CD8 + T cells continue to accumulate with progressing age and can occupy up to 50% of the total memory CD8 + T compartment [13]. In humans, expansion of CMV-specific cells is associated with higher all-cause mortality in older adults [14,15].

A prevailing notion in the field is that the accumulation of CMV-specific T cells corresponds to the priming of naïve and/or memory CD8 + T cells during viral reactivation events [6,16,17]. However, recent work has shown that after initial infection with CMV, the virus does not need to replicate or spread from cell to cell to generate memory inflation [18]. Moreover, Loewendorf et al. demonstrated that memory inflation is maintained in mice after thymectomy, suggesting that recruitment of cells produced after infection is not necessary [17]. Additionally, Dekhtiarenko et al. found that memory inflation is driven by proteasome processing of antigenic epitopes in latently infected cells [19]. Thus, it remains unclear how the inflationary pool of CD8 + T cells is generated and maintained throughout the course of infection.

In the past, T cell immunologists have relied on adoptive cell transfers and bone marrow chimeras to determine which subsets of CD8 + T cells are recruited into the memory pool after infection [6,20–22]. However, because these experimental approaches artificially manipulate the host response, we reasoned that we could obtain new insight into the underlying dynamics of memory inflation by leveraging a fate-mapping system that allows us to track waves of CD8 + T cells produced in the thymus at different periods of life without perturbing immune development or the ongoing infection [23–25]. The advantage of this approach is that we can directly measure the recruitment of endogenous CD8 + T cells during CMV infection with minimal manipulation of the animals. This strategy exploits a TCRδ promoter-driven, tamoxifen-inducible cre (TCRδ-creERT2) to permanently mark, or ‘timestamp,’ CD8 + T cells that are present in the thymus at the time of tamoxifen exposure. The labeled cells can then be tracked in the periphery for the life of the animal. Here, we used our timestamp mice to elucidate how memory inflation is maintained in mice infected with CMV at different stages of life.

## Results

### Neonatal exposure to CMV drives memory inflation of CD8 + T cells

**Fig 1:**
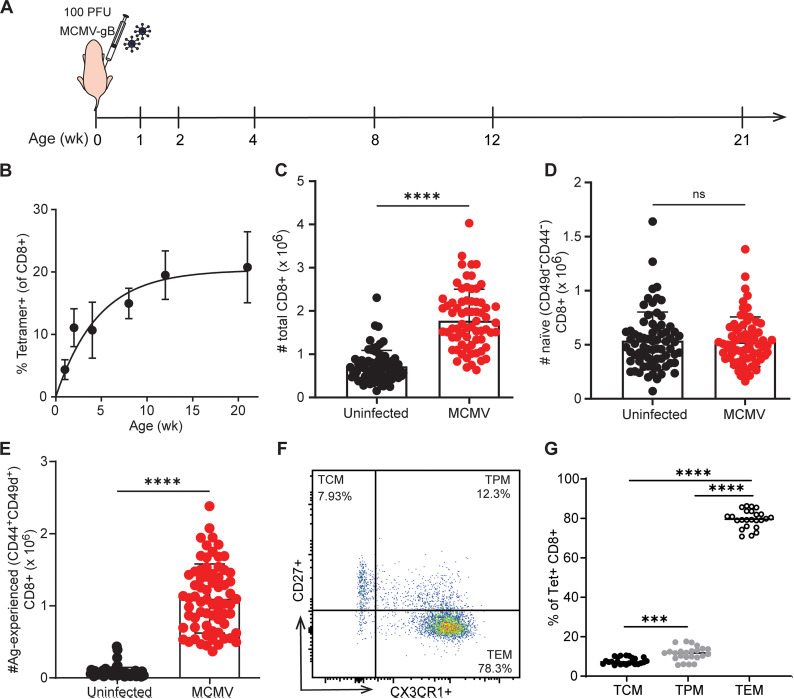
Neonatal exposure to CMV drives a robust memory inflation response. (A) Schematic of experimental design. Newborn mice were infected with MCMV-gB at birth. CD8 + T cells were isolated from the spleen at 1, 2, 4, 8, 12, and 21 weeks post birth. (B) CD8 + T cells were stained with gB tetramer-specific cells and examined by flow cytometry (N = 4–10 mice). At 21 weeks, the total number of (C) CD8 + T cells, (D) naïve cells (CD49d- CD44-) and (E) antigen-experienced CD8 + T cells (CD44 + CD49d+) were quantified. Within the tetramer subgates, memory CD8 + T cells were identified as Central Memory (TCM, CD27 + CX3CR1-), Peripheral Memory (TPM, CD27 + CX3CR1+) or Effector Memory (TEM, CD27- CX3CR1+). (F) Representative dot plots of the gating scheme and (G) quantification of each memory subset are represented. For two groups, an unpaired t-test with Mann-Whitney test for correction was performed. For more than two groups, an ordinary one-way ANOVA with Tukey’s multiple comparisons test was performed. Results are shown as mean ± SD or mean only. ***p < 0.001, ****p < 0.0001.

Since CMV infection is common in early life, we chose to study memory inflation in younger animals [2]. However, less is known about the process of memory inflation after neonatal infection, so we sought to determine how the numbers and phenotype of antigen-specific CD8 + T cells change after MCMV infection in early life. For these studies, we used a recombinant strain of MCMV that expresses the HSV-1 gB-8p peptide (MCMV-gB), which leads to an overwhelmingly dominant gB-specific CD8 + T cell response [26,27], allowing us to monitor the antigen-specific CD8 + T cell response by tracking a single epitope. Mice were infected with MCMV-gB at birth to model congenital infections, as previously described [28,29], while uninfected control mice were given PBS (Fig 1A). CD8 + T cells were collected from the spleen at 1, 2, 4, 8, 12, and 21 weeks of age and stained with antibodies and tetramer (Kb:gB^498-505^) to phenotype the cells at each timepoint. We found that antigen-experienced CD8 + T cells (CD49d+CD44+) accumulated within the CD8 + T cell compartment; this accumulation began to level off at ~60% of the total CD8 + T cells by >5 weeks post infection (S1A Fig). We also measured the number of tetramer positive cells and found that ~20% of CD8 + T cells were gB-specific at 12 weeks of age (Fig 1B). Thus, newborns infected with MCMV mount a robust memory inflation response.

Previous work has shown that memory inflation does not come at the expense of the naïve T cell compartment [30,31]. However, these studies were performed in HCMV-positive adults. Thus, we investigated whether the expansion of memory cells in neonatal-infected mice impacts the number of naïve CD8 + T cells (CD49d-CD44-). To test this, we bled mice and examined the number and phenotype of CD8 + T cells at 16 wks of age. We observed an increase in the total number of cells in the bulk CD8 + T cell compartment (Fig 1C). However, neonatal infection with MCMV did not alter the number of naïve CD8 + T cells. Instead, the observed increase was due to the significantly larger numbers of antigen-experienced CD8 + T cells (Fig 1D-E). These findings are consistent with previous reports, suggesting recruitment of naïve T cells is minimally required for memory inflation [17,32].

We next determined the phenotype of the inflating memory CD8 + T cells. Memory CD8 + T cells can be classified as central memory (CX3CR1- CD27+), peripheral memory (CX3CR1 + CD27+), or effector memory (CX3CR1 + CD27-) [33,34]. At 21 weeks post infection, the antigen-experienced CD8 + T cell pool was comprised of ~60% central memory, ~ 30% effector memory, and ~8% peripheral memory (S1B-C Fig). In contrast, the tetramer positive CD8 + T cell pool was comprised of ~8% central memory, ~ 80% effector memory, and ~12% peripheral memory (Fig 1F-G). Additionally, tetramer+ cells expressed significantly more KLRG1 and significantly less CD62L and CD122 (S1D-G Fig). The phenotype of inflating memory CD8 + T cells in neonatal-infected mice is consistent with previous reports in adult-infected mice, which show that memory inflation leads to an expansion of antigen-specific CD8 + T cells with a terminally differentiated phenotype [6]. Collectively, our data demonstrate that neonatal infection elicits an inflating memory pool that exhibits many of the key features observed in adult infection.

### Neonatal memory inflation is comprised of T cells produced closest to the time of infection

An important question is whether neonatal memory inflation is maintained into adulthood by a self-renewing pool or by recruitment of newly produced CD8 + T cells [35]. To address this question, we used our fate-mapping ‘timestamp’ mouse model to mark CD8 + T cells produced at different times after infection. This mouse model is based on the TCRδ-CreER strain [36,37]. The TCRδ gene is expressed by all T cells in the thymus at the double negative (DN) and double positive (DP) stages of development but is excised in αβ T cells during their transition to the single positive (SP) stage of thymopoiesis [38,39]. Thus, by crossing the TCRδ-creERT2 mice with a reporter strain that has a loxP-flanked ‘stop’ cassette upstream of Zsgreen in the Rosa26 locus (R26R^TdTomato^), we can excise the ‘stop’ cassette and permanently label, or ‘timestamp’, a wave of αβ T cells made in the thymus only during the time of tamoxifen exposure. The wave of labeled cells can then be tracked in the periphery for the life of the animal and be used to understand how the developmental layers of the CD8 + T cell compartment respond differently to infection and environmental factors. Indeed, we recently used this strain to examine how CD8 + T cell development is altered in mice raised in a “clean” (SPF) versus a “dirty” (pet shop-exposed) environment [23,24].

**Fig 2:**
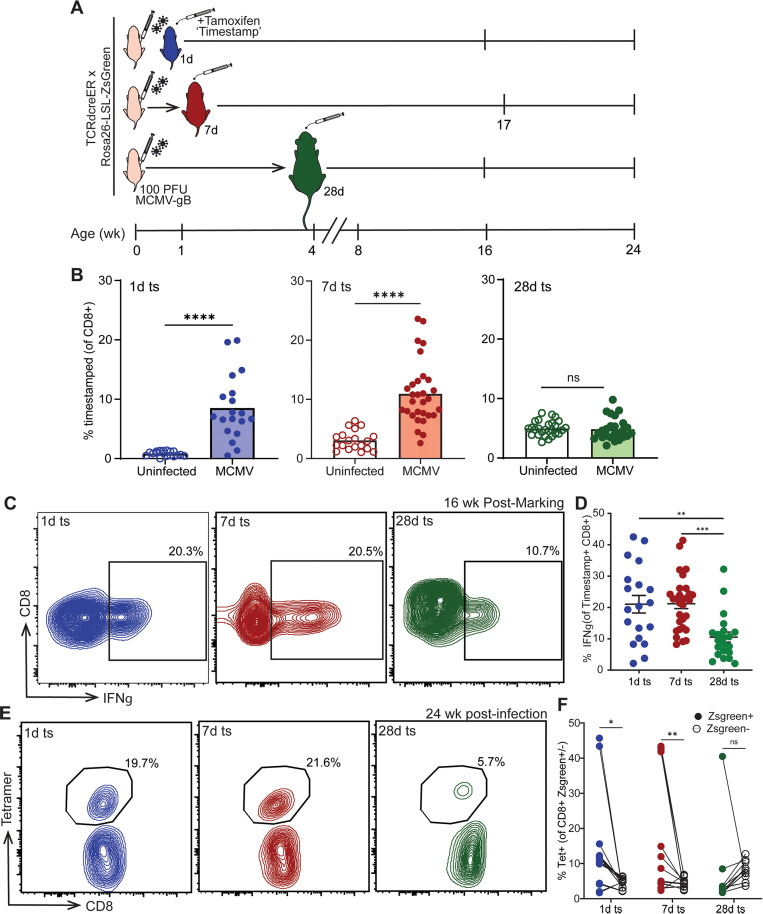
Neonatal CMV infection recruits and maintains CD8 + T cells closest to thymic egress into the memory inflation response. (A) Experimental schematic. Newborn mice were infected with MCMV-gB at birth. Uninfected mice were injected with PBS as control. Mice were given tamoxifen at 1 day, 7 days, or 28 days post birth to ‘timestamp’ CD8 + T cells with a Zsgreen fluorescent tag. (B) Mice were bled at 16–17 weeks post birth, and circulating CD8 + T cells were examined for the percentage marked (ZsGreen+) by flow cytometry (N = 6–24 mice). At 16–17 wks post birth, CD8 + T cells from the spleen were enriched and gB peptide stimulation with BFA was performed for 4 hours (N = 19–30 mice). (C) Representative contour plots of CD8 + IFNγ+ cells and (D) statistical analysis of IFNγ production. Spleens collected at 24 weeks post birth were stained for antigen-specific tetramer+ cells (N = 15–20 mice). (E) Representative contour plots and (F) paired statistical analysis of tetramer+ CD8 + T cells within the timestamp+ and timestamp- populations in the spleen. For two groups, an unpaired t-test with Mann-Whitney test for correction was performed. For more than two unpaired groups, an ordinary one-way ANOVA with Tukey’s multiple comparisons test was performed. For paired group analysis, a two-way RM ANOVA with Bonferroni correction was performed. Results are shown as mean ± SD or mean only. **p < 0.01, ****p < 0.0001.

Since CD8 + T cells from early life are poor at forming memory, it is possible that the inflating pool is sustained by cells produced later in life [40]. On the other hand, neonatal CD8 + T cells persist into adulthood and respond more quickly than adult-derived CD8 + T cells, which may enable them to preferentially respond to viral antigens presented during the latent phase [25,40–42]. To distinguish between these possibilities, we infected timestamp mice at birth with 100 PFU of MCMV-gB. We then administered tamoxifen at 1, 7, and 28 days post birth to ‘mark’ different developmental layers of CD8 + T cells (Fig 2A) [25]. To determine whether the early developmental layers of CD8 + T cells are expanded in adulthood, we bled the mice at 16–17 weeks post infection and compared the relative numbers of cells in infected mice to those found in uninfected controls at 1d, 7d, and 28d (Fig 2A). Interestingly, the 1d marked cells increased from ~1% of the bulk CD8 + T cell compartment in uninfected mice to ~9% in infected mice (Fig 2B, S2A-B Fig). The 7d marked cells increased from ~3% in uninfected mice to ~11% in infected mice (Fig 2B, S2A-B Fig). In the cells made at 28d, there was no significant difference in the percentage of marked cells between uninfected and infected animals (Fig 2B, S2A-B Fig). These data indicate that early life CMV infection can lead to long-term expansion of fetal- and neonatal-derived CD8 + T cells.

In addition, we asked whether the expansion of cells labeled at 1d and 7d was driven by an accumulation of antigen-specific CD8 + T cells. For this question, we bled mice at 16 wks post marking to control for the time the marked cells had been in the periphery undergoing post-thymic maturation. We restimulated the CD8 + T cells with gB peptide and assessed their ability to produce IFNγ using intracellular flow cytometry. The 1d and 7d marked cells restimulated with gB peptide made higher levels of IFNγ (Fig 2C-D). In contrast, fewer 28d marked cells produced IFNγ, which is likely explained by the lower numbers of gB tetramer positive cells in this population (Fig 2C-D). To assess functionality between 1d, 7d, and 28d marked cells, we plotted matched samples of % IFNy+ against the % tetramer + . We observed that the 1d marked cells had an R^2^ = 0.9797, indicating they are highly functional compared to the 7d and 28d marked cells, which had poor correlation (R^2^ = 0.2398, 0.06243 respectively). Additionally, we measured intracellular TNFα and perforin and found that the 1d and 7d marked cells made significantly higher amounts of these inflammatory cytokines and cytolytic molecules (S2D-E Fig).

To confirm that differences in IFNγ production are driven by altered numbers of antigen-specific CD8 + T cells, we collected spleens at 24 wks post birth and examined the percentage of 1d, 7d, and 28d marked cells within the tetramer positive population. Among gB-specific CD8 + T cells, ~ 20% were composed of 1d marked cells and another ~22% were composed of 7d marked cells, while 28d marked cells only constituted ~6% (Fig 2E, S2F Fig). To confirm that what we found in tetramer positive cells was not due to a difference in the magnitude of the memory inflation response, we compared the percentage of tetramer positive cells between Zsgreen timestamped cells and non-timestamped Zsgreen negative cells. As expected, the 1d and 7d marked cells were overrepresented in the tetramer positive population compared to Zsgreen cells (Fig 2F). However, there was no difference between the 28d marked cells and Zsgreen cells, indicating that they were not expanded compared to the unmarked population (Fig 2F). Thus, CMV-specific cells made closest to the time of infection significantly expand and make up a larger proportion of the total CD8 + T cell compartment.

Previous reports have demonstrated that infection of neonatal mice with CMV can lead to prolonged periods of viral replication in organs such as the salivary glands, where virus can be detected for ~5 months post infection in neonates (adults fall below the limit of detection by ~3 months) [28,43,44]. To assess whether our findings hold during periods of ‘true latency,’ we infected mice at birth with 100 PFU of MCMV-gB and administered tamoxifen at 56d (8 wks) and 175d (25 wks) of age (S3A Fig). We allowed the 56d and 175d marked cells to mature to 16 wks post marking, long after the time that replicating virus is detectable in neonates (S3A Fig). We observed no difference in the percentage of 56d or 175d marked cells between uninfected and MCMV-infected mice (S3B-E), which is consistent with the 28d marked cells we examined (Fig 2B, S2A-B). We then performed a gB-peptide restimulation on our CD8 + T cells 16 wks post marking and observed almost no detectable IFNy production in our 56d or 175d marked CD8 + T cells compared to the 1d, 7d, and 28d marked T cells (S3F-G). These findings indicate that newly produced CD8 + T cells are not recruited into the memory inflation response during the latent stage of CMV infection.

Collectively, our data show that T cells exported from the thymus closest to the time of infection are preferentially recruited into the memory inflation response after neonatal infection. These newly produced T cells are known as recent thymic emigrants (RTEs), and they correspond to CD8 + T cells that have been in the periphery for less than 3 wks (in mice) [45]. RTEs exhibit increased expression of integrins (VLA-4, α4β7) and lower amounts of immune functionality (IFNγ, TNFα, IL-2) compared to their mature counterparts during acute infection [46–49]. However, in our neonatal persistent infection model, these RTEs also persist for long periods of time within the host and retain functionality.

### CD8 + recent thymic emigrants preferentially acquire an effector memory phenotype

We next sought to determine whether the phenotype of CD8 + T cells during memory inflation corresponds to their time of production. The ‘bulk’ timestamp population was examined for T cell differentiation based on their expression of CD44 and CD62L. The vast majority of 1d stamped (95%) and 7d stamped (85%) cells exhibited a more terminally differentiated phenotype (CD44 + CD62L-) (S4A-B Fig). In contrast, the 28d stamped CD8 + T cells were comprised of fewer terminally differentiated cells and were the only marked population that contained significant numbers of naïve (CD44-CD62L+) and central or virtual memory (CD44 + CD62L+) cells (S4A-B Fig).

**Fig 3:**
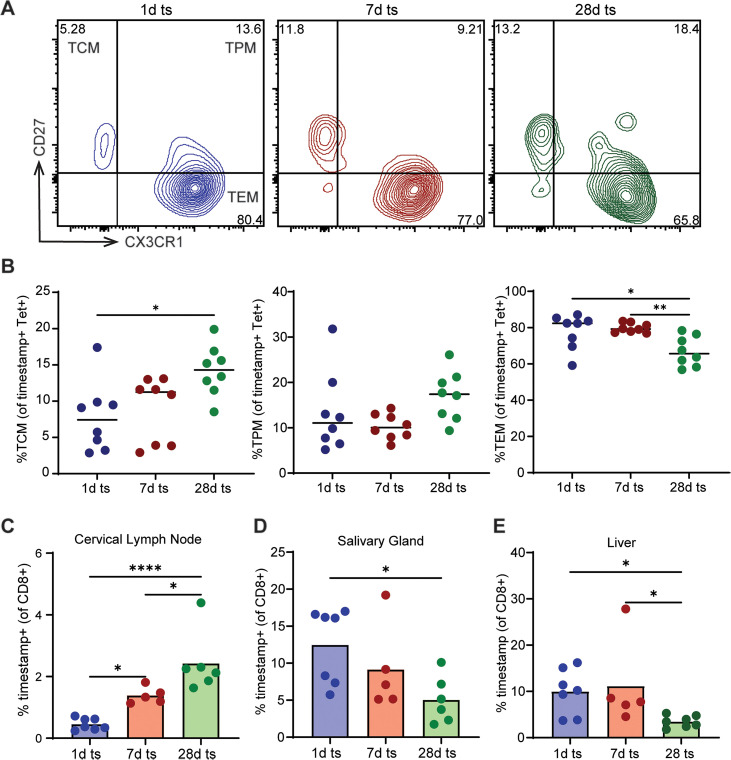
Age of the CD8 + T cells recruited into the memory inflation response dictates phenotype. Newborn mice were infected with MCMV-gB at birth. Uninfected mice were injected with PBS as control. Mice were given tamoxifen at 1d, 7d, or 28d post birth to ‘timestamp’ CD8 + T cells with a Zsgreen fluorescent tag. Spleens were collected at 24 wks post birth, and CD8 + T cells were phenotyped by flow cytometry. (A) Representative density plot of CD27 vs. CX3CR1 within the tetramer+ subgates, where memory CD8 + T cells were identified as Central Memory (TCM, CD27 + , CX3CR1-), Peripheral Memory (TPM, CD27 + , CX3CR1+), or Effector Memory (TEM, CD27-, CX3CR1+). (B) Quantification of TCM, TPM, and TEM phenotypes of 1d, 7d, and 28d timestamped mice within the tetramer+ population (N = 15–20 mice). (C) Quantification of the percentage of timestamped CD8 + T cells within different tissues, as indicated. One-way ANOVA with Tukey’s multiple comparisons test was performed. Results are shown as mean. *p < 0.05, **p < 0.01.

We also used CD27 and CX3CR1 to identify different subsets of memory cells within the antigen-specific (tetramer+) CD8 + T cell pool. Memory T cells with a central memory phenotype (CD27 + CX3CR1-) can travel between circulation and lymphatics, while more terminally differentiated peripheral (CD27 + CX3CR1+) and effector (CD27- CX3CR1+) memory cells circulate in peripheral tissues [16,33]. The antigen-specific 1d and 7d marked cells preferentially gave rise to effector memory cells, while the 28d marked cells exhibited a modest bias towards central memory cells (Fig 3A-B). We also obtained similar results by phenotyping the cells producing IFNγ upon restimulation with peptide. The IFNγ-producing cells in the 1d and 7d marked population exhibited more of an effector memory phenotype, while those in the 28d population exhibited more of a central memory phenotype (S5A-B Fig).

The altered phenotypes of 1d, 7d, and 28d marked cells raised the question of whether the differentially marked T cells localize to different anatomical sites. For example, CX3CR1- (TCM) cells are skewed towards patrolling lymphoid tissue, whereas CX3CR1+ (TPM/TEM) cells are preferentially found in non-lymphoid tissues [33]. We found that 28d marked cells were more significantly enriched in lymphoid tissue (e.g., cervical lymph node) and less abundant in peripheral organs (e.g., salivary gland and liver) than the 1d marked cells (Fig 3C-E). The 7d marked cells exhibited a localization pattern in between the 1d and 28d marked cells; there were more 7d marked cells in the cervical lymph node compared to 1d marked cells, but there were also more 7d marked cells in the liver compared to 28d marked cells. Together, this data suggests that RTEs produced closest to the time of infection have an enhanced capacity to form effector memory cells that migrate to peripheral organs, whereas RTEs produced during the latent stage of infection are biased towards becoming central memory cells and localize to the lymphoid tissue.

### Adult RTEs are preferentially recruited into the inflating pool during later CMV infections

**Fig 4:**
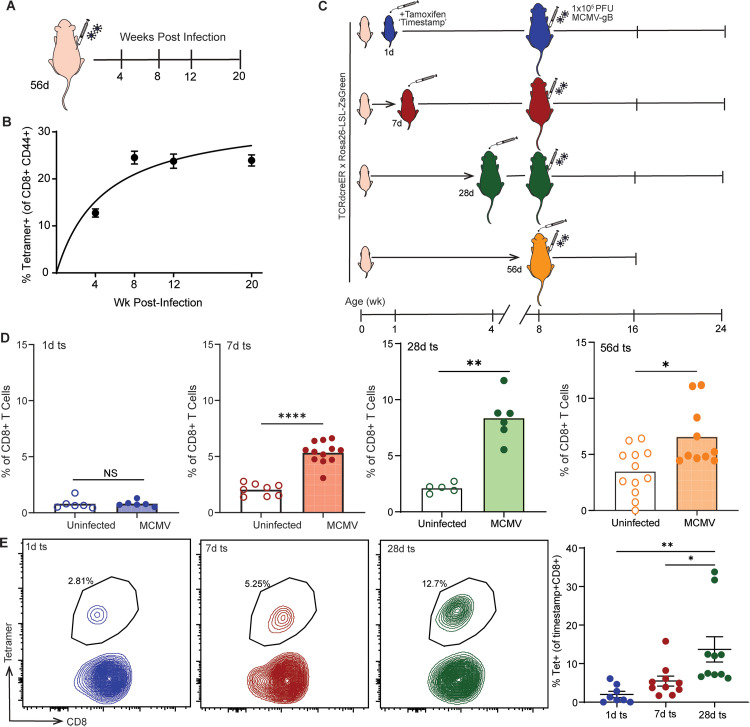
Adult CMV infection recruits and maintains CD8 + T cells closest to thymic egress into the memory inflation response. (A) Adult mice were infected with MCMV-gB at 56d post birth, and spleens were collected at 4, 8, 12, and 20 wks post infection. (B) Quantification of tetramer+ CD8 + T cells. (C) Experimental schematic. Briefly, adult mice were infected with MCMV-gB at 56 days post-birth. Uninfected mice were injected with PBS as control. Mice were given tamoxifen at 1d, 7d, 28d, and 56d post birth to ‘timestamp’ CD8 + T cells with a Zsgreen fluorescent tag. Mice were bled at 16 wks post birth, and circulating ZsGreen+ CD8 + T cells were examined by flow cytometry. (D) The percentage of CD8 + T cells that are ZsGreen+ positive (N = 5–12 mice). (E) Spleens were collected from adults at >24 wks post birth, and CD8 + T cells were stained for gB tetramer. Representative contour plots and percentage of tetramer+ CD8 + T cells in the timestamped population. For two groups, an unpaired t-test with Mann-Whitney test for correction was performed. Results are shown as mean. *p < 0.05, ***p < 0.001, ****p < 0.0001.

The above data did not resolve whether preferential recruitment of RTEs into the CMV response was because 1) 1d marked cells are intrinsically more responsive to MCMV than 28d cells; 2) 1d marked cells were RTEs at the time of infection; or 3) there is less antigen present at the time 28d cells are produced. To distinguish between these possibilities, we infected mice as adults with MCMV-gB and measured the memory inflation response (Fig 4A). Similar to our neonatal MCMV infection model, we confirmed that memory inflation occurred and leveled off at ~25% of the CD8 + T cell pool by 8 wks post infection (Fig 4B, Fig 1B). Additionally, we measured the amount of MCMV virus present between our neonatal- and adult-infected groups. Salivary glands and spleens were collected at 1 wk, 4 wks, and 8 wks post infection for each group, and copy number of MCMV viral gene IE1 was quantified by qPCR and normalized to genomic DNA. In the salivary gland, we observed slightly higher (although not statistically significant) amounts of viral DNA in the neonatally infected mice at 1 and 4 wks; however, by 8 wks, there were equivalent levels of viral DNA between the neonatal- and adult-infected groups (S6A Fig). In the spleen, we saw a reverse trend, where neonatal-infected mice had less viral DNA compared to adult-infected mice at 1, 4, and 8 wks post infection, although the differences were not statistically significant (S6B Fig). Together, these data argue against the possibility that more RTEs are recruited into the response because of higher levels of viral DNA during early stages of infection.

To look at the recruitment of RTEs into the memory inflation response during adult MCMV infection, mice were timestamped at 1d, 7d, 28d, and 56d post birth and infected with 1x10^5^ PFU of MCMV-gB at 8 wks (56d) post birth (Fig 4C). At the time of infection, the 28d marked cells had been present in the periphery for approximately 2 weeks, and the 56d marked cells were undergoing thymic selection and exiting the thymus, making these groups the most recently produced population of CD8 + T cells (i.e., RTEs) [36]. To control for the age of the host, we bled the mice at 16 wks post birth to match the timepoint examined after neonatal infection and measured the percentage of cells marked at different ages. Interestingly, there was no significant difference in the percentage of total 1d stamped cells between uninfected and adult MCMV-infected animals (Fig 4D, S6C-D Fig). However, there was a significant increase in the percentage of 7d, 28d, and 56d marked cells in adult MCMV-infected animals, with the 28d and 56d being the most pronounced population (Fig 4D, S6C-D Fig). We also examined the differentiation and activation status of the marked cells and observed less differentiated (CD44 + CD62L+) cells in the 1d and 7d marked populations and more terminally differentiated (CD44 + CD62L-) cells in the 28d marked population (S7A-B Fig).

Similar to the neonatal-infected mice, we bled the adult-infected mice at 16 wks post infection and performed a gB peptide restimulation of the CD8 + T cells to measure antigen-specific production of IFNγ. We found that CD8 + T cells marked at 56d made significantly higher amounts of IFNy in response to peptide compared to both 7d and 28d marked cells (S7C Fig). We also collected spleens at 24 wks of age and measured the antigen-specific tetramer response in the adult-infected mice. The 28d marked cells had a significant increase in the percentage of tetramer positive cells compared to both 1d and 7d marked cells (Fig 4E). In fact, only ~3% of the 1d marked cells were tetramer positive, while ~5–6% of the 7d and ~13% of the 28d marked cells contributed to the antigen response (Fig 4E).

Up to this point, we utilized the MCMV-gB strain because it elicits a dominant and easily trackable antigen-specific CD8 + T cell response. However, the wildtype (Smith) strain of MCMV has many acute (M45, M57) and inflating epitopes (IE3, M38, M139), raising the possibility that our observations were skewed by this ‘artificial’ dominant epitope of MCMV-gB. To address this possibility, we first measured the percentage of CD8 + T cells that were antigen-experienced by staining for tetramers against gB, M45, M139, IE3, and M38 in adult mice infected with MCMV-Smith or MCMV-gB (both 1x10^5^ PFU) at 8 wks of age. At 6 wks post infection, we observed an equivalent percentage of tetramer+ CD8 + T cells, although gB-specific CD8 + T cells dominated the response after MCMV-gB and M38-specific CD8 + T cell dominated the response after MCMV (Smith) (S8A Fig). To determine whether RTEs were being recruited into the inflating response following infection with wildtype MCMV, we administered tamoxifen to timestamp mice at 1d, 7d, and 28d and infected each group with MCMV-Smith at 8 wks of age. At 4 wks post infection, we observed no significant difference in 1d or 7d marked CD8 + T cells between uninfected and MCMV-Smith infected mice (S8B Fig). However, at 4 wks, 28d marked cells were significantly increased in MCMV-Smith mice (S8B Fig). When we stained with WT tetramers (M45, M139, IE3, M38), we observed that the 28d marked CD8 + T cells were comprised of significantly more M38 + antigen-experienced cells than the 1d and 7d marked cells (S8C Fig). Thus, the preferential recruitment of CD8 + T cells produced closest to the time of infection and their bias towards becoming effector memory is neither a unique attribute of neonatal infections nor dependent upon a particular strain of virus, but instead appears to be a common feature of the CD8 + T cell response to CMV infection in later life.

### Post-thymic maturation is a key determinant of T cell recruitment during CMV infection

Up to this point, we have shown that RTEs are preferentially recruited into the memory inflation pool in both neonatal and adult infections. While these experiments provide important information about the CD8 + T cell response to infection at different stages of life, there are several key variables not controlled for in each infection scenario. For example, in the adult-infected mice, it is possible that the 1d marked cells are not efficiently recruited into the response not because they have undergone more post-thymic maturation, but because they are simply outcompeted by the 28d marked cells, which are derived from a different progenitor and likely express more diverse TCRs [25,40,50,51].

**Fig 5:**
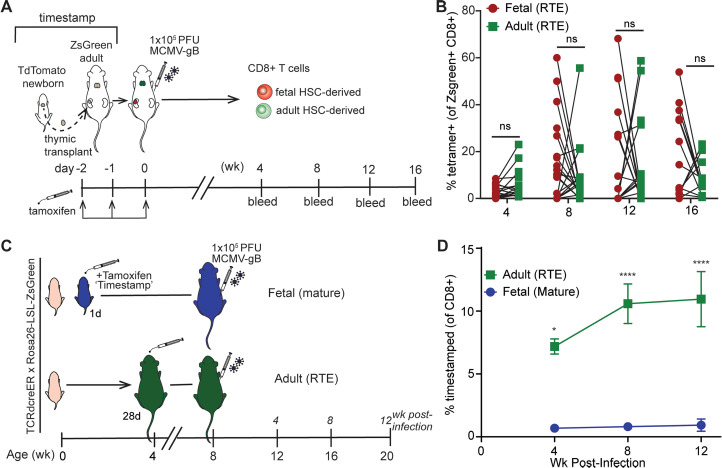
Recent thymic emigrants are preferentially recruited into the memory inflation response regardless of developmental origin. (A) Experimental schematic. Newborn thymuses were collected from TdTomato+ timestamp reporter mice and surgically transplanted under the kidney capsule into adult (>8 wk) ZsGreen+ timestamp reporter mice. Mice were administered tamoxifen for 3 d and then infected with MCMV-gB two days after marking. At 4, 8, 12, and 16 wks post infection, mice were bled and the CD8 + T cell memory response was measured by flow cytometry. (B) The percentage of CD8 + cells that were ZsGreen/TdTomato marked cells (N = 13–21 mice). A two-way repeated measures ANOVA with Bonferroni test correction was performed. Results are shown as pair-wise comparison, where each connected point is one mouse. (C) Experimental schematic. Mice were administered tamoxifen at 1d to mark the ‘fetal’ layer or 28d post birth to mark the ‘adult’ layer. All mice were infected at 56d post birth and bled 4, 8, and 12 wks post infection. (D) The percentage of CD8 + T cells from 1d or 28d marked layers. A two-way ANOVA with Bonferroni test correction was performed. *p < 0.05, ***p < 0.001, ****p < 0.0001.

To control for the age of the cells and compare how RTEs of different developmental origins respond to infection in the same environment, we performed a thymic transplant experiment where the thymus from a fetal (TdTomato) timestamp donor mouse was engrafted under the kidney capsule of an adult (ZsGreen) timestamp mouse (Fig 5A). Thus, when tamoxifen is administered, it will mark CD8 + T cells of fetal (red) and adult (green) origin at the same time and allow us to directly compare fetal RTEs vs. adult RTEs in the same (adult) environment. If developmental origin is a key determinant, we would expect to see either the fetal or adult RTE population get preferentially recruited into the adult response. If post-thymic maturation is the major factor, the fetal and adult RTEs should behave similarly. On day 5 after thymic transplant, the recipient mice were infected with MCMV-gB and bled every 4 wks to measure expansion of the marked fetal and adult cells (Fig 5A). We found that there was no statistical difference between the expansion of both fetal and adult CD8 + T cells at 4, 8, 12, or 16 wks post infection (Fig 5B). At 16 wks post infection, we collected spleens from the mice to examine the andphenotype of donor cells their response to gB peptide restimulation. We found no significant difference in the production of IFNγ upon peptide restimulation, consistent with our data showing no difference in tetramer response (S9A Fig). Fetal donor cells had a more differentiated phenotype, which was high for KLRG1, low for CD62L, and significantly skewed towards effector memory (CX3CR1 + CD27-) compared to adult donor cells (S9B-E Fig). These findings extend our previous work showing that fetal-derived CD8 + T cells have a more terminally differentiated phenotype after acute infection, indicating the phenomenon also occurs during persistent infection [52].

To validate the significance of our thymic transplant findings, we used our model from Fig 4 and examined the fetal and adult layer of CD8 + T cells in mice infected as adults with MCMV-gB at the same timepoints as those used in the transplant studies (Fig 5C). In this scenario, all fetal-marked cells would be “mature”, while the adult-marked cells would be closest to the RTE population (Fig 5C). As expected, only the adult RTE cells expanded at 4, 8, and 12 wks post infection, while the fetal mature cells failed to expand during the course of infection (Fig 5D). Taken together, this data shows that developmental origin does not dictate the response to memory inflation, but that the age of the cell is a key determining factor.

**Fig 6:**
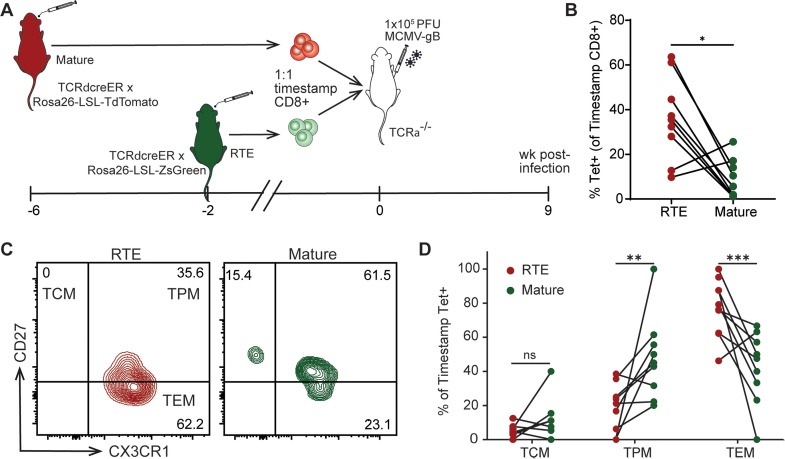
RTE-derived memory cells adopt a terminally differentiated phenotype. (A) Experimental schematic. Adult ZsGreen+ mice were marked with tamoxifen 6 wks prior to harvest, while adult Ai9 TdTomato+ mice were marked with tamoxifen 2 wks prior to harvest. CD8 + T cells were isolated from the spleen. A 1:1 ratio of ZsGreen+ and TdTomato+ CD8 + T cells were pooled and co-transferred into adult TCRα^-/-^ animals. The next day, recipient animals were infected with MCMV-gB. Mice were bled at 9 wks post infection, and the CD8 + T cell response was measured by flow cytometry. (B) The percentage of ZsGreen or TdTomato-marked CD8 + T cells that were Tetramer+ (N = 9). (C) Representative 2-way FACS plot of CD27 vs. CX3CR1. (D) The percentage of tetramer+ timestamped CD8 + T cells that adopted a central memory (TCM), peripheral memory (TPM), or effector memory (TEM) phenotype. A paired t-test with Wilcoxon matched pairs signed rank test performed for correction. Results are shown as pair-wise comparison, where each connected point is one mouse. *p < 0.05, ***p < 0.001, ****p < 0.0001.

Another variable not controlled for in earlier experiments was the precursor number. For example, it is possible that we saw more RTEs respond to adult infection because there were more 28d marked cells than 1d marked cells at the time of infection. One way to experimentally test this possibility is to directly compare equal numbers of adult (28d marked) cells in the same environment that only differ in maturation (RTEs vs. mature cells). For these studies, we again turned to our timestamp model. In this case, ZsGreen+ timestamp mice were given tamoxifen 6 wks prior to infection, making the marked CD8 + T cells mature at the time of harvest. A second group of adult TdTomato+ timestamp mice were given tamoxifen 2 wks prior to infection, making the marked CD8 + T cells RTEs at the time of harvest. Marked cells from both donor populations were harvested and combined in a 1:1 ratio and transferred into TCRα^-/-^ animals that were then infected the following day (Fig 6A). We found that the TdTomato+ (RTE) population was more readily recruited into the antigen-specific tetramer response than the mature ZsGreen+ (mature) population at 9 wks post infection (Fig 6B). Consistent with our previous observations, RTEs recruited into the memory inflation response more readily adopted a terminally differentiated effector memory phenotype (Fig 5C-D). Altogether, our findings indicate that RTEs are a major source of cells recruited into the memory inflating pool and are retained for the life of the animal.

## Discussion

Previous work has suggested that CD8 + T cell responses are maintained over the course of persistent infection because they are replenished by a constant pool of short-lived effector cells [6,53,54]. Conceptually, one could hypothesize that the short-lived effectors replacing the pool are recruited from new RTEs that are constantly produced during latent infection. However, as we demonstrate, this is not the case. Specifically, we did not observe an equal recruitment of CD8 + T cells produced at different stages of life or ongoing recruitment of cells during persistent infection. Instead, the CMV-specific memory pool was maintained by cells produced closest to when the host was first infected. This led us to propose that there is a critical window for CD8 + T cells to be recruited into the inflating pool, which corresponds to how recently they were exported from the thymus at the time of infection. Our findings show that this is indeed the case, and indicate that the effector CD8 + T cells forming the inflationary pool have a long half-life in peripheral tissues [55].

Our results are significant for several reasons. First, memory inflation is typically studied in animals infected in adulthood [6,18,56,57]. Here, we examined memory inflation in early life, when the host is more susceptible to CMV [58,59]. Despite many age-related differences between the immune systems of neonatal and adult mice [60,61], we found that RTEs are preferentially recruited into the memory pool regardless of the timing of infection. Second, there is a general assumption that less cell recruitment happens during the latter stage of infection because CMV becomes latent in non-hematopoietic cells, which are poor at priming naïve CD8 + T cells [32,62–65]. However, we found that RTEs are preferentially recruited into the memory inflating pool even when exposed to the same amount of antigen/inflammation as their more mature counterparts, indicating that cell-intrinsic differences also determine which cells are recruited into the response.

Although the underlying basis for why RTEs are preferentially recruited into the CD8 + T cell response after persistent viral infection is not clear, several possibilities are worth considering. First, RTEs are more biased towards becoming short-lived effectors than mature CD8 + T cells, even when responding to infection in the same host [47,48]. The enhanced propensity for RTEs to undergo effector cell differentiation may allow them to more rapidly fill the memory inflation pool. Second, RTEs express higher levels of VLA-4 than mature cells, which enhances their ability to migrate into various tissues [46]. The increased ability of RTEs to gain access to peripheral tissues may enable them to encounter more CMV antigens than mature cells and undergo memory inflation. Third, RTEs have a superior ability to respond to low-affinity antigens [46]. Thus, it is possible that RTEs are preferentially recruited into the inflating response because they can respond to CMV antigens that are not recognized by mature cells. Future experiments are required to examine the relative contribution of these possibilities.

A possible explanation for the differences we observed in recruitment of timestamped CD8 + T cells is precursor frequency. Although 1d and 7d cells are the only ones present during the acute phase of neonatal infection, CD8 + T cells made at all stages of life are present in the adult infection model. We previously published a study estimating the total numbers of 1d, 7d, and 28d marked CD8 + T cells in naïve mice at 56 days of age [24]. While there were slightly less 1d marked cells, we found no significant difference between 7d and 28d marked cells in uninfected mice at 56d of age, suggesting that the increase in 28d marked cells after adult infection is not simply explained by differences in the numbers of cells present within each developmental layer prior to infection [24]. However, one potential limitation of our current study is that we did not enumerate the exact number of epitope-specific precursors that were present within each developmental layer at the time of infection. Thus, we cannot rule out the possibility that certain epitope-specific precursors are preferentially made at distinct stages of life.

A novel finding from our study is that timing of infection dictates the types of CD8 + T cells that are maintained in the memory pool. Whereas infections in early life expand the neonatal layer of CD8 + T cells, infections in adulthood promote an increase in adult CD8 + T cells. Thus, an important question is, are there consequences to having a larger number of neonatal cells in the adult memory pool? Previous work has indicated that CMV infection contributes significantly to immune variation in the human population [30,66,67]. Whether timing of infection explains some of the variability in outcomes among CMV patients remains an open question.

Our work also sheds light on competing models of memory inflation. Work from the Oxenius lab and others suggest that KLRG1- TCF1 + central memory-like CD8 + T cells maintain the inflationary pool of T cells during CMV [54,68,69]. The Buchholz lab demonstrated that these CD8 + T cell central memory precursors control the magnitude of the memory inflation response [70]. Other evidence from the Klenerman lab shows that a self-proliferating pool of peripheral memory CD8 + T cells replenishes the inflationary pool after CMV infection [16]. Recent work supports the idea that viral reactivation is not a driving force of memory inflation, as CMV viral genes are transcribed stochastically during latency [42]. However, Gerlach et al. demonstrated that peripheral memory CD8 + T cells are highly plastic and exist in a transient state where they can revert back to a central memory phenotype or terminally differentiate into an effector memory phenotype, which could explain why there are currently multiple models of how the inflationary pool is maintained [33]. Importantly, our studies may be be viewed alongside current models. For example, our results support the prevailing idea that long-lived populations maintain effectors. However, we show that this population is selectively recruited from RTEs present at the time of infection (rather than from the total CD8 + T cell pool).

Lastly, our work provides new insight into age-related differences in the CD8 + T cell response to persistent viral infections. Neonates and lymphoreplete individuals have a larger and more robust pool of RTEs in their CD8 + T cell compartments [60,71]. It is interesting to speculate whether memory inflation occurs at a more rapid rate because of this larger RTE pool. Humans seropositive for CMV have a diverse percentage of CMV-specific CD8 + T cells. In one study, the memory CD8 + T cell pool specific to HCMV of seropositive individuals ranged from <2% to >40% (average of 10.2%) [10]. It will be important to determine whether the size of the RTE pool at the time of infection has a direct impact on the rate, size, and phenotype of the inflationary pool within individuals. This may have particular implications for individuals acquiring CMV after HSCT or other lymphodepleting therapy, which may affect RTE frequency at the time of infection. Our data showing that RTEs are preferentially recruited into the response means that the age of the cell present at the time of infection will need to be taken into account when developing future strategies to boost immunity against persistent viral infections at different stages of life.

## Materials and methods

### Ethics statement

All experiments in this study were conducted in accordance with the recommendations in the Guide for the Care and Use of Laboratory Animals of the National Institutes of Health and protocols reviewed and approved by the Institutional Animal Care and Use Committee at Cornell University.

### Animals

C57BL/6NCR mice were purchased from Charles River. gBT-I TCR transgenic mice (specific for the HSV-1 glycoprotein gB498–505 peptide, SSIEFARL) were provided by Dr. Janko Nikolich-Zugich (University of Arizona, Tucson, AZ). ZsGreen (Ai6), TdTomato (Ai9), TCRδCre-ERT2 and TCRα^-/-^ mice were purchased from Jackson Laboratories. Male and female mice were used for all experiments unless otherwise specified. All results are pooled experiments from different batches of mice.

### Timestamping mice

Mice were generated by breeding TCRδCre-ERT2 with ZsGreen or TdTomato reporter mice. To mark 1d (fetal) T cells, 2.5 mg of tamoxifen was administered to dams by oral gavage, 3 times in 12 hour increments. Pups received the tamoxifen through lactation. To mark 7d (neonatal) T cells, pups were administered tamoxifen 0.25 mg by oral gavage. To mark 28d (adult) T cells mice were given 2.5 mg of tamoxifen by oral gavage. To mark 56d and 175d (mature adult) T cells mice were given 5 mg of tamoxifen for 3 consecutive days by oral gavage.

### MCMV infection

Recombinant MCMV (Smith strain) expressing the MHC class I-restricted CTL epitope HSV gB498–505 (SSIEFARL), designated in the text as MCMV-gB, was provided by Dr. Cicin-Sain (Helmholtz Centre for Infection Research, Germany). The gB-8p peptide sequence was inserted into the MCMV genome at the 3’ end of the MCMV, i.e.,2 gene as previously described [26]. Newborn pups (<24 hours postpartum) were infected intraperitoneally (i.p.) with 100 plaque forming units (PFU) for the newborn MCMV group of animals. Adult mice were infected i.p. with 1x10^5^ PFU for adult MCMV group of animals.

MCMV-gB and MCMV-Smith viral stocks were propagated in M2-10B4 cells (CRL-1972, ATCC) as previously described [72]. All media used was RP-10: 10% FBS, 2mM L-glutamine, 50U/mL Penicillin/Streptomycin, 1x 2-Mercaptoethanol, RPMI 1640. M2-10B4 cells were grown to 80–90% confluence in T175 flasks, ∼1.33 × 10^5^ PFU of MCMV stock was used to inoculate the confluent monolayer such that cells are infected at an MOI of ∼0.01 PFU/cell. Infected flasks were incubated at 37°C in a 5% CO2 for 4 hours, tilting the dishes every 30 min to ensure even distribution of virus inoculum. Fresh RP-10 media prewarmed to 37°C was added to the flasks and incubate the dishes for 3–5 days at 37°C in a 5% CO2 incubator, until all cells in a monolayer exhibit a cytopathic effect. Cells were collected with cell scraper and centrifuged for 30 min at 2000 × g in 4°C. Supernatant was collected as stored in -80°C as crude viral stocks. Final concentration of virus was determined by plaque assay as previously published [73]. M2-10B4 cells were grown in 6-well plates until ~80–90% confluent. 1% (w/v) Methylcellulose was made by autoclaving 500mL dH20 with 10g methylcellulose (Sigma), once cool mixed 1:1 with 20% FBS 2X RPMI (Gibco). Crude viral stocks were diluted in PBS from 10^-1^ to 10^-8^ and the 6 most diluted concentrations were used to infect each well of the 6-well plates containing M2-10B4 cells. Virus was allowed to infect cells for 3 hours, then media was aspirated and prewarmed 1% Methylcellulose was added to each well. After 5 days cells were washed with PBS, stained with 0.1% (w/v) Crystal Violet (Sigma) and washed with dH20. Once plates dried the number of plaques were counted and PFU/mL was back calculated.

### Flow cytometry

Mouse antibodies for CD4 (Gk1.5), CD8a (53-6.7), CD27 (LG.3A10), CD44 (IM7), CD49d (R1-2), CD62L (MEL-14), CD122 (TM-B1), CX3CR1 (SA011F11), TCRβ (H57-597), TCRγδ (eBioGL3), Perforin (S16009A), TNFα (MP6-XT22), IFNγ (XMG1.2), KLRG1 (2F1), and CD62L (MEL-14) were purchased from BD Bioscience, BioLegend or ThermoFisher Scientific. Fixable viability dye eFluor 780 was purchased from TheremoFisher Scientific. Biotinylated monomers against gB, M45, M38, M139, and IE3 peptides were obtained from the National Institutes of Health Tetramer Core Facility and tetramerized with streptavidin-linked fluorophores within our lab. In brief, monomers were thawed to room temperature, 10 aliquots of desired streptavidin conjugates were made from either PE or APC respectively. Each aliquot of streptavidin conjugate was added to the monomer every 10 min in the hood making sure to cover the tube in foil to protect from light. After adding each aliquot, the tube was briefly and gentle vortexed and put on the rocker in the cold room. Tetramers were then tested at concentrations of 1:100, 1:500 and 1:1000 for optimal staining conditions.

Whole blood was lysed for red blood cells by addition of dH20 followed by quickly adding 4x PBS. Lysis was performed twice and then washed twice with MACS solution (1x PBS, 0.5% BSA, 2mM EDTA). Spleens were homogenized using a syringe over a 40 uM filter. CD8 + T cells were bead enriched from the spleens by positive selection with CD8a microbeads (Miltenyi). Spins between each was and resuspension was at 500g for 3 minutes in 96 well plates. Cells were stained in antibody cocktail suspended in Brilliant Stain Buffer (BD Biosciences) and MACS. Cells were stained for 30 minutes without tetramer or 60 minutes with tetramer at 4^o^C in the dark. For surface staining cells were fixed with IC fixation kit (Invitrogen). For intracellular staining cells were permeabilized using Foxp3/Transcription Factor Staining Buffer (Invitrogen). Cells were incubated with intracellular antibodies in Brilliant Stain Buffer (BD Biosciences) and MACS for 30 minutes at 4^o^C in the dark. All cells were resuspended as a final single cell suspension in MACS.

For gating schemes lymphocytes were identified by FSC/SSC. Singlets were gated on FSC/FSH and SSC/SSH. Conventional CD8 + T cells were identified by TCRγδ-/CD4-/TCRβ + /CD8a + . Antigen-specific cells were identified by tetramer staining, timestamp cells were ZsGreen + , true naïve CD8 + T cells were gated as CD49d-/CD44-/CD122-, virtual memory CD8 + T cells were gated as CD49d-/CD44 + /CD122+ and true memory (TMEM) CD8 + T cells were gated as (CD49d + /CD44+). Within the TMEM population central memory (TCM) was identified as CD27 + CX3CR1-, peripheral memory (TPM) as CD27 + CX3CR1+ and effector memory (TEM) as CD27- CX3CR1 + .

All experiments were run on BD FACSymphony A3, BD FACSymphony A5 or Accuri C6. Data was then analyzed on FlowJo Version 10.

### In vitro stimulation

CD8 + T cells from whole blood or spleen were stimulated with 1 x 10^-7^ M of SSIEFARL peptide with 1.5 µg/ml Brefeldin A (Millipore Sigma) for 4 hours at 37^O^C resuspended in RP-10 media (RPMI 1640, 10% fetal bovine serum, 1x L-glutamine, 1x penicillin/streptomycin).

### Cell counts

ZsGreen reporter mice were bred for cell enumeration. Mice were either left uninfected or infected with MCMV at D0 or D56 same as experimental groups. Spleens, cervical lymph nodes and thymus were collected and processed into a single cell suspension. Total cell counts were enumerated by a MoxiZ (ORFLO) and the absolute number of CD8 + T cells were back calculated by the percentage of CD3 + CD8a+ cells on flow cytometry.

### Co-transfer experiment

ZsGreen timestamp mice at 6 weeks of age were marked with tamoxifen for 3 days in 24-hour increments, 6 weeks prior to harvesting the spleen. TdTomato timestamp mice at 4 weeks of age were marked with tamoxifen for 3 days in 24-hour increments, 2 weeks prior to harvesting the spleen. CD8 + T cells were isolated from ZsGreen+ and TdTomato+ spleens at the same time. Cells were enumerated and a 1:1 of 200k ZsGreen+ and 200k TdTomato+ CD8 + T cells were pooled and transferred into TCRα^-/-^ animals by intravenous injection. The next day TCRα^-/-^ recipients were infected with MCMV-gB and subsequently retro-orbitally bled 9 weeks post-infection.

### Thymic transplants

Transplants were conducted as previously described with modification [25]. Thymus lobes were isolated from 1d TdTomato+ timestamp mice. The lobes were placed surgically under the kidney capsule of a 7 week old ZsGreen+ timestamp recipient mouse. To mark the thymocytes, 5 mg of tamoxifen was administered by oral gavage for 3 days post-surgery in 24 hour increments. Mice were infected with MCMV-gB at 5 days post-surgery and bled retro-orbitally at 4, 8 and 12 weeks post-infection. Spleens were harvested at 16 weeks post-infection for downstream analysis.

### Viral quantification

Mice were infected with MCMV-gB at either D0-D1 post-birth (neonatal MCMV-gB) or at 8 weeks of age (adult MCMV-gB). Salivary glands and spleens were collected from infected mice at 1, 4 and 8 weeks post-infection. DNA was isolated from the collected organs by the DNeasy Blood and Tissue Kit (Qiagen) according to the manufacturer instructions. Total genomic DNA was quantified by nanodrop. Quantitative polymerase chain reaction (qPCR) was performed utilizing the PerfeCTa SYBR Green FastMix, Low ROX (Quantabio). Samples were run on the Applied Biosystems ViiA 7 Teal-Time PCR system utilizing the Fast 2-Step Cycling of initial denaturatioin (95^o^C, 30s), 40 PCR cycles (95^o^C, 5s), collect data at end of extension step (60^o^C, 30s). MCMV IE1 viral DNA was quantified by standard curve quantification. The standard curve was generated by taking a known starting concentration of purified MCMV-gB and subsequent serial dilution. Concentration of viral DNA was quantified by standard curve utilizing ViiA™ 7 Software. Quantified IE1 viral DNA was normalized to input genomic DNA measured by nanodrop. Primers to MCMV IE1 were obtained by Integrated DNA Technologies (IDT).

### Statistical analysis

All error bars are represented as mean plus or minus standard deviation or standard error of the mean. For comparison between two groups an Unpaired t test was used; if the standard deviation was not equal a Welch’s correction was applied. All statistics were performed in GraphPad Prism Version 9. For comparison between three or more groups an One-way or Two-way ANOVA was used for appropriate test correction (Bonferroni, Tukey) as indicated in each figure legend. The regression line shown in Figs 1A, 4A, and S1A was an One phase exponential association as defined by Y = Ymax*(1-exp(-K*X)) where the line starts at zero and ascends to Ymax with a rate constant K, half time is 0.69/K.

## Supporting information

S1 Fig Neonatal infection with MCMV undergoes inflation of ‘antigen-experienced’ CD8 + T cells. **Newborn mice were infected with MCMV-gB at birth.** CD8 + T cells were isolated from the spleen at 1, 2, 4, 8, 12 and 21 weeks post-birth. (A) CD8 + T cells were stained for CD44 and CD49d to measure ‘antigen-experienced’ cell by flow cytometry (N = 4–10 mice). (B) CD8 + T cells within the CD44 + CD49d + sub-gate were stained for CD27 vs CX3CR1 to distinguish memory phenotype (Central Memory [TCM, CD27 + CX3CR1-], Peripheral Memory [TPM, CD27 + CX3CR1+], Effector Memory [TEM, CD27- CX3CR1+]). Representative 2-way FACS plot of CD27 vs CX3CR1. (C) Quantification of TCM, TPM and TEM CD8 + T cells within the CD44 + CD49d + sub-gate. (D-F) Quantification of KLRG1, CD62L, and CD122 expression between tetramer+ and tetramer- cells. (F-G) Representative FACS histograms for KLRG1, CD62L, and CD122. For (C), an ordinary One-way ANOVA with Tukey’s multiple comparisons test was performed. For (D, E and F) paired t-tests with Wilcoxon matched-pairs signed rank test for correction were performed. Results are shown as mean ± SD or mean only. **p < 0.01, ****p < 0.0001.
(PDF)

S2 Fig CD8 + T cells produced closest to neonatal infection are preferentially recruited into memory inflation response and are highly functional. Newborn TCRδcreERT2 x ZsGreen mice were infected with MCMV-gB at birth. Uninfected mice were injected with PBS as control. Mice were given tamoxifen at 1 day, 7 days, or 28 days post-birth to ‘timestamp’ CD8 + T cells with a Zsgreen fluorescent tag. Mice were bled at 16–17 weeks to measure percentage of CD8 + T cells Zsgreen + . Representative contour plots of (A) uninfected or (B) neonatally infected timestamp mice showing the ‘timestamped’ population in each group. Spleens were collected at 24 weeks post-birth. (C) comparison of tetramer+ and IFNγ + ZsGreen+ T cells with R^2^ values reported from simple linear regression. (D) Percentage of total timestamp CD8 + T cells that make TNFα. (E) Percentage of total timestamp CD8 + T cells that make Perforin. (F) Percentage of tetramer+ within Zsgreen+ CD8 + T cells. For D-F, an ordinary One-way ANOVA with Tukey’s multiple comparisons test was performed. Results are shown as mean ± SEM.
(PDF)

S3 Fig Adult cells are not efficiently recruited into the inflating response to neonatal MCMV during the period of latency. (A) Experimental schematic. Newborn TCRδcreERT2 x ZsGreen mice were infected with MCMV-gB at birth. Uninfected mice were injected with PBS as control. Mice were given tamoxifen at 56 or 175 days post-birth to ‘timestamp’ CD8 + T cells with a Zsgreen fluorescent tag. Mice were bled at 16 weeks post-marking. (B-C) representative contour plots of Zsgreen timestamping in the CD8 + populations of uninfected (B) or neonatally infected (C) mice. (D-E) Quantification of timestamped CD8 + T cells in 56 day (D) and 175 day (E) timestamp mice. (F-G) CD8 + T cells from the blood of 1d, 7d, 28d, 56d, and 175d marked mice at 16 weeks post-marking were gB peptide stimulated with BFA for 4 hours. Cells were then intracellularly stained for effector IFNγ. (F) Quantification of IFNγ production in indicated ‘timestamped’ groups. (G) Representative contour plots of IFNγ production in 56d (top) and 175d (bottom) timestamped cells. For D and E, Unpaired t-tests with Mann-Whitney test for correction were performed. Results are shown as mean only. For F) an one-way ANOVA with Tukey’s multiple comparisons test was performed. *p < 0.05 **p < 0.01, ****p < 0.0001.
(PDF)

S4 Fig Following neonatal infection, RTEs adopt a terminally differentiated phenotype. Newborn TCRδcreERT2 x ZsGreen mice were infected with MCMV-gB at birth. Uninfected mice were injected with PBS as control. Mice were given tamoxifen at 1 day, 7 days, or 28 days post-birth to ‘timestamp’ CD8 + T cells with a Zsgreen fluorescent tag. (A) Representative 2-way plot of CD44 vs CD62L on total timestamp CD8 + T cells. (B) Percentage of total timestamp CD8 + T cells that adopted an CD44 vs CD62L phenotype. For B, a two-way ANOVA with Tukey’s multiple comparisons test was performed. Results are shown as mean ± SEM.
(PDF)

S5 Fig Functional antigen-specific RTEs primarily become effector memory cells. Newborn TCRδcreERT2 x ZsGreen mice were infected with MCMV-gB at birth. Uninfected mice were injected with PBS as control. Mice were given tamoxifen at 1 day, 7 days, or 28 days post-birth to ‘timestamp’ CD8 + T cells with a Zsgreen fluorescent tag. (A) Representative density plot of CD27 vs CX3CR1 within the IFNγ + sub gates where memory CD8 + T cells were identified as Central Memory (TCM, CD27 + CX3CR1-), Peripheral Memory (TPM, CD27 + CX3CR1+) or Effector Memory (TEM, CD27- CX3CR1+). (B) Quantification of TCM, TPM and TEM phenotype of 1d, 7d and 28d timestamped mice within the IFNγ+ population (N = 8 mice). For statistical test of more than two-groups, an ordinary One-way ANOVA with Tukey’s multiple comparisons test was performed. Results are shown as mean ± SD or mean only. ***p < 0.001, ****p < 0.0001.
(PDF)

S6 Fig Cells made early in life are not efficiently recruited into the CD8 + T cell response to adult MCMV infection. Quantification of viral replication in neonatal (black) or adult (red) MCMV-gB infection at indicated times post infection as measured by IE1 copy number within the (A) salivary gland and (B) spleen. (C-D) Adult mice were infected with MCMV-gB at 56 days post-birth. Uninfected mice were injected with PBS as control. Mice were given tamoxifen at 1 day, 7 days, 28 days or 56 days post-birth to ‘timestamp’ CD8 + T cells with a Zsgreen fluorescent tag. Mice were bled at 16 weeks post-birth and circulating Zsgreen+ CD8 + T cells were examined by flow cytometry. (C) Representative contour plots of uninfected 1d, 7d, 28d and 56d timestamp mice. (D) Representative contour plots of infected 1d, 7d, 28d and 56d timestamp mice. A two-way ANOVA with Bonferroni test correction was performed.
(PDF)

S7 Fig CD8 + T cells produced closest to adult MCMV infection (RTEs) adopt a more terminally differentiated and antigen-experienced phenotype. Adult timestamp mice were infected with MCMV-gB at 56 days post-birth and spleens were collected from adults at >24 weeks post-birth. CD8 + T cells were stained for CD44 vs CD62L to determine differentiation status. (A) Representative 2-way plot of CD44 vs CD62L on total timestamp CD8 + T cells. (B) Quantification of CD44 vs CD62L on total timestamp CD8 + T cells. (C-D. CD8 + T cells from the blood were given gB peptide stimulation with BFA was performed for 4 hours (N = 5–12 mice per group) (C) representative contour plots of and (D) statistical analysis of IFNγ production. For B) a two-way ANOVA with Bonferroni test correction was performed. For C) an ordinary One-way ANOVA with Tukey’s multiple comparisons test was performed. Results are shown as mean ± SEM. *p < 0.05, ***p < 0.001.
(PDF)

S8 Fig RTEs are preferentially recruited into the naturally inflating CD8 + T cell pool during MCMV-Smith infection. Adult mice were infected with MCMV-Smith or MCMV-gB (as indicated) at 56 days post-birth. (A) Proportion of responding CD8 + T cells pool to indicated T cell epitope, measured 6 wk following infection, N = 4–6. (B) Adult timestamp mice were infected with MCMV-Smith at 56 days post-birth. Uninfected mice were injected with PBS as control. Mice were given tamoxifen at 1 day, 7 days, 28 days or 56 days post-birth to ‘timestamp’ CD8 + T cells with a Zsgreen fluorescent tag. Mice were bled at 4 weeks post infection and ZsGreen+ percentage was measured (N = 5). (C) Percentage of epitope-specific, tetramer+ Zsgreen+ T cells at 4 weeks post infection (N = 5). (D) Representative contour plots showing M38-specific (inflating) ZsGreen+ CD8 + T cells at 4 weeks post infection. For A and C) a two-way ANOVA with Bonferroni test correction was performed. For B) an unpaired t-test with Mann-Whitney test for correction was performed.
(PDF)

S9 Fig Fetal and Adult RTEs become terminally-differentiated TEM cells. Newborn thymuses were collected from TdTomato+ timestamp reporter mice and surgically transplanted under the kidney capsule into adult (>8 week) Zsgreen+ timestamp reporter mice. Mice were administered tamoxifen for 3 days and then infected with MCMV-gB two days after marking. CD8 + T cell phenotypes were measured by flow cytometry at 16 week post infection. (A) Quantification of % of timestamped CD8 + T cells producing IFNγ. (B) Quantification of % of timestamped CD8 + T cells that express KLRG1. (C) Quantification of % of timestamped CD8 + T cells that express CD62L. (D) Quantification of memory CD8 + T cells identified as Central Memory (TCM, CD27 + CX3CR1-), Peripheral Memory (TPM, CD27 + CX3CR1+) or Effector Memory (TEM, CD27- CX3CR1+). (E) Representative contour plots showing previously indicated memory populations in fetal- (top) and adult-(bottom) derived RTEs. For A-C) an paired t-test with Wilcoxon matched pairs signed rank test performed for correction. For D) A two-way repeated measures ANOVA with Bonferroni test correction was performed. *p < 0.05, ***p < 0.001, ****p < 0.0001.
(PDF)

S1 Data Raw dataset. Values used to build graphs, generate means, standard error, standard deviations and statistical analysis.
(XLSX)

## References

[1] Manicklal S, Emery VC, Lazzarotto T, Boppana SB, Gupta RK. The “silent” global burden of congenital cytomegalovirus. Clin Microbiol Rev. 2013;26(1):86–102. doi: 10.1128/CMR.00062-12 23297260 PMC3553672

[2] Cannon MJ, Schmid DS, Hyde TB. Review of cytomegalovirus seroprevalence and demographic characteristics associated with infection. Rev Med Virol. 2010;20(4):202–13. doi: 10.1002/rmv.655 20564615

[3] Pass RF, Little EA, Stagno S, Britt WJ, Alford CA. Young children as a probable source of maternal and congenital cytomegalovirus infection. N Engl J Med. 1987;316(22):1366–70. doi: 10.1056/NEJM198705283162203 3033505

[4] Cannon MJ, Hyde TB, Schmid DS. Review of cytomegalovirus shedding in bodily fluids and relevance to congenital cytomegalovirus infection. Rev Med Virol. 2011;21(4):240–55. doi: 10.1002/rmv.695 21674676 PMC4494736

[5] Nikolich-Žugich J, et al. Advances in cytomegalovirus (CMV) biology and its relationship to health, diseases, and aging. Geroscience. 2020;42(2):495–504. 32162210 10.1007/s11357-020-00170-8 PMC7205956

[6] Snyder CM, Cho KS, Bonnett EL, van Dommelen S, Shellam GR, Hill AB. Memory inflation during chronic viral infection is maintained by continuous production of short-lived, functional T cells. Immunity. 2008;29(4):650–9. doi: 10.1016/j.immuni.2008.07.017 18957267 PMC2583440

[7] Karrer U, Sierro S, Wagner M, Oxenius A, Hengel H, Koszinowski UH, et al. Memory inflation: continuous accumulation of antiviral CD8+ T cells over time. J Immunol. 2003;170(4):2022–9. doi: 10.4049/jimmunol.170.4.2022 12574372

[8] Looney RJ, Falsey A, Campbell D, Torres A, Kolassa J, Brower C, et al. Role of cytomegalovirus in the T cell changes seen in elderly individuals. Clin Immunol. 1999;90(2):213–9. doi: 10.1006/clim.1998.4638 10080833

[9] Wertheimer AM, et al. Aging and cytomegalovirus infection differentially and jointly affect distinct circulating T cell subsets in humans. J Immunol. 2014;192(5):2143–55. 24501199 10.4049/jimmunol.1301721 PMC3989163

[10] Sylwester AW, Mitchell BL, Edgar JB, Taormina C, Pelte C, Ruchti F, et al. Broadly targeted human cytomegalovirus-specific CD4+ and CD8+ T cells dominate the memory compartments of exposed subjects. J Exp Med. 2005;202(5):673–85. doi: 10.1084/jem.20050882 16147978 PMC2212883

[11] Bolinger B, Sims S, O’Hara G, de Lara C, Tchilian E, Firner S, et al. A new model for CD8+ T cell memory inflation based upon a recombinant adenoviral vector. J Immunol. 2013;190(8):4162–74. doi: 10.4049/jimmunol.1202665 23509359 PMC3672979

[12] Barber DL, Wherry EJ, Masopust D, Zhu B, Allison JP, Sharpe AH, et al. Restoring function in exhausted CD8 T cells during chronic viral infection. Nature. 2006;439(7077):682–7. doi: 10.1038/nature04444 16382236

[13] Jergović M, Contreras NA, Nikolich-Žugich J. Impact of CMV upon immune aging: facts and fiction. Med Microbiol Immunol. 2019;208(3–4):263–9. doi: 10.1007/s00430-019-00605-w 31004198 PMC6635032

[14] Savva GM, Pachnio A, Kaul B, Morgan K, Huppert FA, Brayne C, et al. Cytomegalovirus infection is associated with increased mortality in the older population. Aging Cell. 2013;12(3):381–7. doi: 10.1111/acel.12059 23442093

[15] Aiello AE, Chiu Y-L, Frasca D. How does cytomegalovirus factor into diseases of aging and vaccine responses, and by what mechanisms? Geroscience. 2017;39(3):261–71. doi: 10.1007/s11357-017-9983-9 28624868 PMC5505887

[16] Gordon CL, Lee LN, Swadling L, Hutchings C, Zinser M, Highton AJ, et al. Induction and Maintenance of CX3CR1-Intermediate Peripheral Memory CD8+ T Cells by Persistent Viruses and Vaccines. Cell Rep. 2018;23(3):768–82. doi: 10.1016/j.celrep.2018.03.074 29669283 PMC5917822

[17] Loewendorf AI, Arens R, Purton JF, Surh CD, Benedict CA. Dissecting the requirements for maintenance of the CMV-specific memory T-cell pool. Viral Immunol. 2011;24(4):351–5. doi: 10.1089/vim.2010.0140 21721929 PMC3154399

[18] Snyder CM, Cho KS, Bonnett EL, Allan JE, Hill AB. Sustained CD8+ T cell memory inflation after infection with a single-cycle cytomegalovirus. PLoS Pathog. 2011;7(10):e1002295. doi: 10.1371/journal.ppat.1002295 21998590 PMC3188546

[19] Dekhtiarenko I, Ratts RB, Blatnik R, Lee LN, Fischer S, Borkner L, et al. Peptide Processing Is Critical for T-Cell Memory Inflation and May Be Optimized to Improve Immune Protection by CMV-Based Vaccine Vectors. PLoS Pathog. 2016;12(12):e1006072. doi: 10.1371/journal.ppat.1006072 27977791 PMC5158087

[20] Billingham RE, Silvers WK, Wilson DB. Further studies on adoptive transfer of sensitivity to skin homografts. J Exp Med. 1963;118(3):397–420. doi: 10.1084/jem.118.3.397 14078000 PMC2137648

[21] Miller TA. Transfer of immunity to Ancylostoma caninum infection in pups by serum and lymphoid cells. Immunology. 1967;12(2):231–41. 6020124 PMC1409282

[22] Vezys V, Masopust D, Kemball CC, Barber DL, O’Mara LA, Larsen CP, et al. Continuous recruitment of naive T cells contributes to heterogeneity of antiviral CD8 T cells during persistent infection. J Exp Med. 2006;203(10):2263–9. doi: 10.1084/jem.20060995 16966427 PMC2118117

[23] Tabilas C, Iu DS, Daly CWP, Yee Mon KJ, Reynaldi A, Wesnak SP, et al. Early microbial exposure shapes adult immunity by altering CD8+ T cell development. Proc Natl Acad Sci U S A. 2022;119(49):e2212548119. doi: 10.1073/pnas.2212548119 36442114 PMC9894172

[24] Reynaldi A, Smith NL, Schlub TE, Tabilas C, Venturi V, Rudd BD, et al. Fate mapping reveals the age structure of the peripheral T cell compartment. Proc Natl Acad Sci U S A. 2019;116(10):3974–81. doi: 10.1073/pnas.1811634116 30765525 PMC6410819

[25] Smith NL, et al. Developmental origin governs CD8( ) T cell fate decisions during infection. Cell. 2018;174(1):117-130.e14. 10.1016/j.cell.2018.05.029 29909981

[26] Dekhtiarenko I, et al. The context of gene expression defines the immunodominance hierarchy of cytomegalovirus antigens. J Immunol. 2013;190(7):3399–409. 23460738 10.4049/jimmunol.1203173

[27] Venturi V, Nzingha K, Amos TG, Charles WC, Dekhtiarenko I, Cicin-Sain L, et al. The Neonatal CD8+ T Cell Repertoire Rapidly Diversifies during Persistent Viral Infection. J Immunol. 2016;196(4):1604–16. doi: 10.4049/jimmunol.1501867 26764033 PMC4744528

[28] Bantug GRB, Cekinovic D, Bradford R, Koontz T, Jonjic S, Britt WJ. CD8+ T lymphocytes control murine cytomegalovirus replication in the central nervous system of newborn animals. J Immunol. 2008;181(3):2111–23. doi: 10.4049/jimmunol.181.3.2111 18641350 PMC4161464

[29] Brizić I, Hiršl L, Britt WJ, Krmpotić A, Jonjić S. Immune responses to congenital cytomegalovirus infection. Microbes Infect. 2018;20(9–10):543–51. doi: 10.1016/j.micinf.2017.12.010 29287989 PMC6019571

[30] Samson LD, et al. Limited effect of duration of CMV infection on adaptive immunity and frailty: insights from a 27-year-long longitudinal study. Clin Transl Immunol. 2020;9(10):e1193. 10.1002/cti2.1193 PMC7586993 33133599

[31] van den Heuvel D, et al. Cytomegalovirus- and Epstein-Barr virus-induced T-cell expansions in young children do not impair naive T-cell populations or vaccination responses: the Generation R study. J Infect Dis. 2016;213(2):233–42. 26142434 10.1093/infdis/jiv369

[32] Torti N, Walton SM, Brocker T, Rülicke T, Oxenius A. Non-hematopoietic cells in lymph nodes drive memory CD8 T cell inflation during murine cytomegalovirus infection. PLoS Pathog. 2011;7(10):e1002313. doi: 10.1371/journal.ppat.1002313 22046127 PMC3203160

[33] Gerlach C, et al. The chemokine receptor cx3cr1 defines three antigen-experienced cd8 t cell subsets with distinct roles in immune surveillance and homeostasis. Immunity. 2016;45(6):1270–84. 27939671 10.1016/j.immuni.2016.10.018 PMC5177508

[34] Böttcher JP, Beyer M, Meissner F, Abdullah Z, Sander J, Höchst B, et al. Functional classification of memory CD8(+) T cells by CX3CR1 expression. Nat Commun. 2015;6:8306. doi: 10.1038/ncomms9306 26404698 PMC4667439

[35] Klenerman P. The (gradual) rise of memory inflation. Immunol Rev. 2018;283(1):99–112. doi: 10.1111/imr.12653 29664577 PMC5947157

[36] Zhang B, Wu J, Jiao Y, Bock C, Dai M, Chen B, et al. Differential Requirements of TCR Signaling in Homeostatic Maintenance and Function of Dendritic Epidermal T Cells. J Immunol. 2015;195(9):4282–91. doi: 10.4049/jimmunol.1501220 26408667 PMC4610875

[37] Zhang B, Jia Q, Bock C, Chen G, Yu H, Ni Q, et al. Glimpse of natural selection of long-lived T-cell clones in healthy life. Proc Natl Acad Sci U S A. 2016;113(35):9858–63. doi: 10.1073/pnas.1601634113 27535935 PMC5024599

[38] Krangel MS, Carabana J, Abbarategui I, Schlimgen R, Hawwari A. Enforcing order within a complex locus: current perspectives on the control of V(D)J recombination at the murine T-cell receptor alpha/delta locus. Immunol Rev. 2004;200:224–32. doi: 10.1111/j.0105-2896.2004.00155.x 15242408

[39] Prinz I, Sansoni A, Kissenpfennig A, Ardouin L, Malissen M, Malissen B. Visualization of the earliest steps of gammadelta T cell development in the adult thymus. Nat Immunol. 2006;7(9):995–1003. doi: 10.1038/ni1371 16878135

[40] Wang J, et al. Fetal and adult progenitors give rise to unique populations of CD8 T cells. Blood. 2016;128(26):3073–82. 28034872 10.1182/blood-2016-06-725366 PMC5201096

[41] Lemmermann NAW, Reddehase MJ. Direct evidence for viral antigen presentation during latent cytomegalovirus infection. Pathogens. 2021;10(6). 10.3390/pathogens10060731 PMC8229173 34200578

[42] Griessl M, Renzaho A, Freitag K, Seckert CK, Reddehase MJ, Lemmermann NAW. Stochastic Episodes of Latent Cytomegalovirus Transcription Drive CD8 T-Cell “Memory Inflation” and Avoid Immune Evasion. Front Immunol. 2021;12:668885. doi: 10.3389/fimmu.2021.668885 33968074 PMC8100209

[43] Reddehase MJ, Balthesen M, Rapp M, Jonjić S, Pavić I, Koszinowski UH. The conditions of primary infection define the load of latent viral genome in organs and the risk of recurrent cytomegalovirus disease. J Exp Med. 1994;179(1):185–93. doi: 10.1084/jem.179.1.185 8270864 PMC2191331

[44] Moulden J, et al. Murine models of central nervous system disease following congenital human cytomegalovirus infections. Pathogens. 2021;10(8). 10.3390/pathogens10081062 PMC8400215 34451526

[45] Cunningham CA, Helm EY, Fink PJ. Reinterpreting recent thymic emigrant function: defective or adaptive? Curr Opin Immunol. 2018;51:1–6. doi: 10.1016/j.coi.2017.12.006 29257954 PMC5943149

[46] Berkley AM, Fink PJ. Cutting edge: CD8 recent thymic emigrants exhibit increased responses to low-affinity ligands and improved access to peripheral sites of inflammation. J Immunol. 2014;193(7):3262–6. 25172492 10.4049/jimmunol.1401870 PMC4170019

[47] Deets KA, Berkley AM, Bergsbaken T, Fink PJ. Cutting Edge: Enhanced Clonal Burst Size Corrects an Otherwise Defective Memory Response by CD8+ Recent Thymic Emigrants. J Immunol. 2016;196(6):2450–5. doi: 10.4049/jimmunol.1502525 26873989 PMC4779721

[48] Makaroff LE, Hendricks DW, Niec RE, Fink PJ. Postthymic maturation influences the CD8 T cell response to antigen. Proc Natl Acad Sci U S A. 2009;106(12):4799–804. doi: 10.1073/pnas.0812354106 19270077 PMC2660772

[49] Houston EG Jr, Higdon LE, Fink PJ. Recent thymic emigrants are preferentially incorporated only into the depleted T-cell pool. Proc Natl Acad Sci U S A. 2011;108(13):5366–71. doi: 10.1073/pnas.1015286108 21402911 PMC3069187

[50] Smith NL, Wissink E, Wang J, Pinello JF, Davenport MP, Grimson A, et al. Rapid proliferation and differentiation impairs the development of memory CD8+ T cells in early life. J Immunol. 2014;193(1):177–84. doi: 10.4049/jimmunol.1400553 24850719 PMC4065808

[51] Rudd BD, Venturi V, Smith NL, Nzingha K, Goldberg EL, Li G, et al. Acute neonatal infections “lock-in” a suboptimal CD8+ T cell repertoire with impaired recall responses. PLoS Pathog. 2013;9(9):e1003572. doi: 10.1371/journal.ppat.1003572 24068921 PMC3771883

[52] Reynaldi A, Smith NL, Schlub TE, Venturi V, Rudd BD, Davenport MP. Modeling the dynamics of neonatal CD8+ T-cell responses. Immunol Cell Biol. 2016;94(9):838–48. doi: 10.1038/icb.2016.47 27142943 PMC5069106

[53] Smith CJ, Venturi V, Quigley MF, Turula H, Gostick E, Ladell K, et al. Stochastic Expansions Maintain the Clonal Stability of CD8+ T Cell Populations Undergoing Memory Inflation Driven by Murine Cytomegalovirus. J Immunol. 2020;204(1):112–21. doi: 10.4049/jimmunol.1900455 31818981 PMC6920548

[54] Welten SPM, Yermanos A, Baumann NS, Wagen F, Oetiker N, Sandu I, et al. Tcf1+ cells are required to maintain the inflationary T cell pool upon MCMV infection. Nat Commun. 2020;11(1):2295. doi: 10.1038/s41467-020-16219-3 32385253 PMC7211020

[55] Baumann NS, Torti N, Welten SPM, Barnstorf I, Borsa M, Pallmer K, et al. Tissue maintenance of CMV-specific inflationary memory T cells by IL-15. PLoS Pathog. 2018;14(4):e1006993. doi: 10.1371/journal.ppat.1006993 29652930 PMC5919076

[56] Santosa EK, et al. Defining molecular circuits of CD8 T cell responses in tissues during latent viral infection. J Exp Med. 2025;222(8). 10.1084/jem.20242078 PMC12087470 40387857

[57] Snyder CM, Loewendorf A, Bonnett EL, Croft M, Benedict CA, Hill AB. CD4+ T cell help has an epitope-dependent impact on CD8+ T cell memory inflation during murine cytomegalovirus infection. J Immunol. 2009;183(6):3932–41. doi: 10.4049/jimmunol.0900227 19692644 PMC2766182

[58] Koontz T, Bralic M, Tomac J, Pernjak-Pugel E, Bantug G, Jonjic S, et al. Altered development of the brain after focal herpesvirus infection of the central nervous system. J Exp Med. 2008;205(2):423–35. doi: 10.1084/jem.20071489 18268036 PMC2271002

[59] Noyola DE, et al. Cytomegalovirus urinary excretion and long term outcome in children with congenital cytomegalovirus infection. Pediatr Infect Dis J. 2000;19(6):505–10. 10877163 10.1097/00006454-200006000-00003

[60] Rudd BD. Neonatal T Cells: A Reinterpretation. Annu Rev Immunol. 2020;38:229–47. doi: 10.1146/annurev-immunol-091319-083608 31928469 PMC7369171

[61] Adkins B, Leclerc C, Marshall-Clarke S. Neonatal adaptive immunity comes of age. Nat Rev Immunol. 2004;4(7):553–64. doi: 10.1038/nri1394 15229474

[62] Munks MW, Rott K, Nesterenko PA, Smart SM, Williams V, Tatum A, et al. Latent CMV infection of Lymphatic endothelial cells is sufficient to drive CD8 T cell memory inflation. PLoS Pathog. 2023;19(1):e1010351. doi: 10.1371/journal.ppat.1010351 36689486 PMC9894547

[63] Reddehase MJ, Lemmermann NAW. Cellular reservoirs of latent cytomegaloviruses. Med Microbiol Immunol. 2019;208(3–4):391–403. doi: 10.1007/s00430-019-00592-y 31011793

[64] Smith CJ, Turula H, Snyder CM. Systemic hematogenous maintenance of memory inflation by MCMV infection. PLoS Pathog. 2014;10(7):e1004233. doi: 10.1371/journal.ppat.1004233 24992722 PMC4081724

[65] Mercer JA, Wiley CA, Spector DH. Pathogenesis of murine cytomegalovirus infection: identification of infected cells in the spleen during acute and latent infections. J Virol. 1988;62(3):987–97. 2828694 10.1128/jvi.62.3.987-997.1988 PMC253658

[66] Breznik JA, Huynh A, Zhang A, Bilaver L, Bhakta H, Stacey HD, et al. Cytomegalovirus Seropositivity in Older Adults Changes the T Cell Repertoire but Does Not Prevent Antibody or Cellular Responses to SARS-CoV-2 Vaccination. J Immunol. 2022;209(10):1892–905. doi: 10.4049/jimmunol.2200369 36426948 PMC9666329

[67] Yan Z, Maecker HT, Brodin P, Nygaard UC, Lyu SC, Davis MM, et al. Aging and CMV discordance are associated with increased immune diversity between monozygotic twins. Immun Ageing. 2021;18(1):5. doi: 10.1186/s12979-021-00216-1 33461563 PMC7812659

[68] Baumann NS, Welten SPM, Torti N, Pallmer K, Borsa M, Barnstorf I, et al. Early primed KLRG1- CMV-specific T cells determine the size of the inflationary T cell pool. PLoS Pathog. 2019;15(5):e1007785. doi: 10.1371/journal.ppat.1007785 31083700 PMC6532941

[69] Gabel M, Baumann NS, Oxenius A, Graw F. Investigating the Dynamics of MCMV-Specific CD8+ T Cell Responses in Individual Hosts. Front Immunol. 2019;10:1358. doi: 10.3389/fimmu.2019.01358 31281313 PMC6595046

[70] Grassmann S, Mihatsch L, Mir J, Kazeroonian A, Rahimi R, Flommersfeld S, et al. Early emergence of T central memory precursors programs clonal dominance during chronic viral infection. Nat Immunol. 2020;21(12):1563–73. doi: 10.1038/s41590-020-00807-y 33106669

[71] Davenport MP, Smith NL, Rudd BD. Building a T cell compartment: how immune cell development shapes function. Nat Rev Immunol. 2020;20(8):499–506. doi: 10.1038/s41577-020-0332-3 32493982 PMC7390700

[72] Brizić I, et al. Cytomegalovirus infection: mouse model. Curr Protoc Immunol. 2018;122(1):e51. 10.1002/cpim.51 PMC6347558 30044539

[73] Lutarewych MA, et al. Propagation and titration of murine cytomegalovirus in a continuous bone marrow-derived stromal cell line (M2-10B4). J Virol Methods. 1997;68(2):193–8. 9389409 10.1016/s0166-0934(97)00126-2

