# Supplementary figures and images for: Recent thymic emigrants preferentially undergo memory inflation after persistent infection
Source: PLoS Pathog. 2025 Jul 28;21(7):e1013382. doi: 10.1371/journal.ppat.1013382 (PMC12316393; doi:10.1371/journal.ppat.1013382)

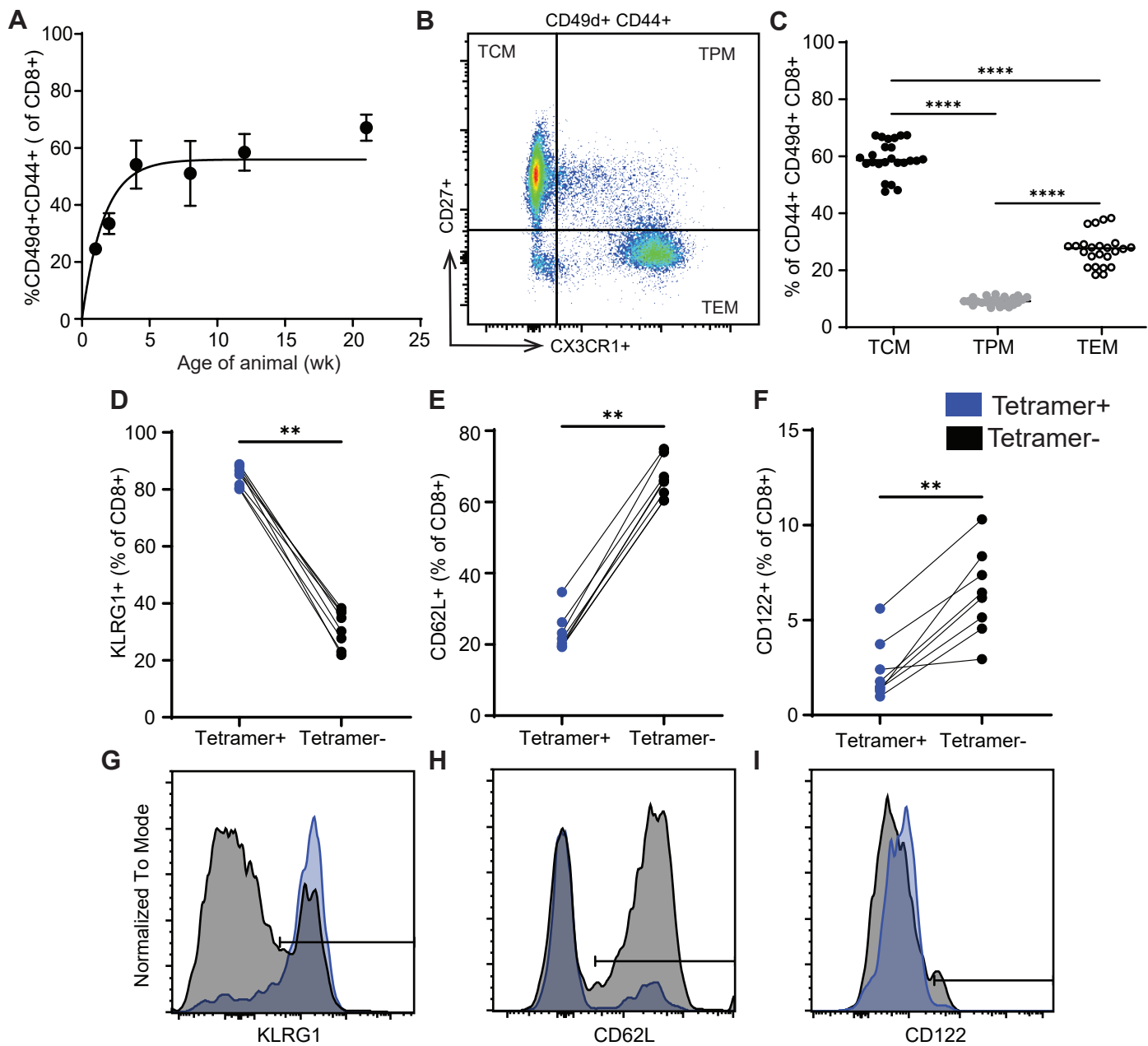

Supplement: S1 Fig — Newborn mice were infected with MCMV-gB at birth. CD8 + T cells were isolated from the spleen at 1, 2, 4, 8, 12 and 21 weeks post-birth. (A) CD8 + T cells were stained for CD44 and CD49d to measure ‘antigen-experienced’ cell by flow cytometry (N = 4–10 mice). (B) CD8 + T cells within the CD44 + CD49d + sub-gate were stained for CD27 vs CX3CR1 to distinguish memory phenotype (Central Memory [TCM, CD27 + CX3CR1-], Peripheral Memory [TPM, CD27 + CX3CR1+], Effector Memory [TEM, CD27- CX3CR1+]). Representative 2-way FACS plot of CD27 vs CX3CR1. (C) Quantification of TCM, TPM and TEM CD8 + T cells within the CD44 + CD49d + sub-gate. (D-F) Quantification of KLRG1, CD62L, and CD122 expression between tetramer+ and tetramer- cells. (F-G) Representative FACS histograms for KLRG1, CD62L, and CD122. For (C), an ordinary One-way ANOVA with Tukey’s multiple comparisons test was performed. For (D, E and F) paired t-tests with Wilcoxon matched-pairs signed rank test for correction were performed. Results are shown as mean ± SD or mean only. **p < 0.01, ****p < 0.0001. (PDF) [file ppat.1013382.s001.pdf]

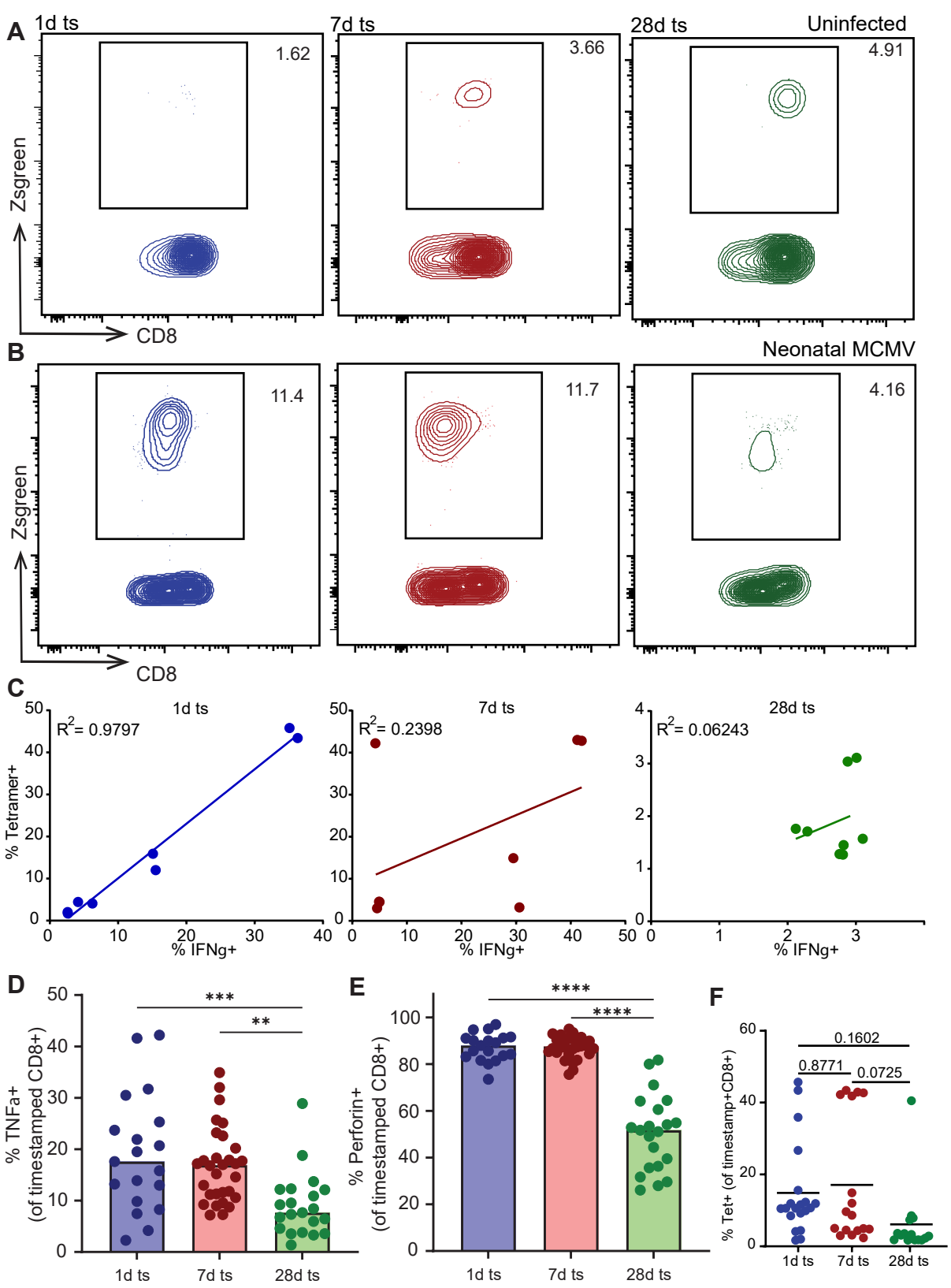

Supplement: S2 Fig — Newborn TCRδcreERT2 x ZsGreen mice were infected with MCMV-gB at birth. Uninfected mice were injected with PBS as control. Mice were given tamoxifen at 1 day, 7 days, or 28 days post-birth to ‘timestamp’ CD8 + T cells with a Zsgreen fluorescent tag. Mice were bled at 16–17 weeks to measure percentage of CD8 + T cells Zsgreen + . Representative contour plots of (A) uninfected or (B) neonatally infected timestamp mice showing the ‘timestamped’ population in each group. Spleens were collected at 24 weeks post-birth. (C) comparison of tetramer+ and IFNγ + ZsGreen+ T cells with R2 values reported from simple linear regression. (D) Percentage of total timestamp CD8 + T cells that make TNFα. (E) Percentage of total timestamp CD8 + T cells that make Perforin. (F) Percentage of tetramer+ within Zsgreen+ CD8 + T cells. For D-F, an ordinary One-way ANOVA with Tukey’s multiple comparisons test was performed. Results are shown as mean ± SEM. (PDF) [file ppat.1013382.s002.pdf]

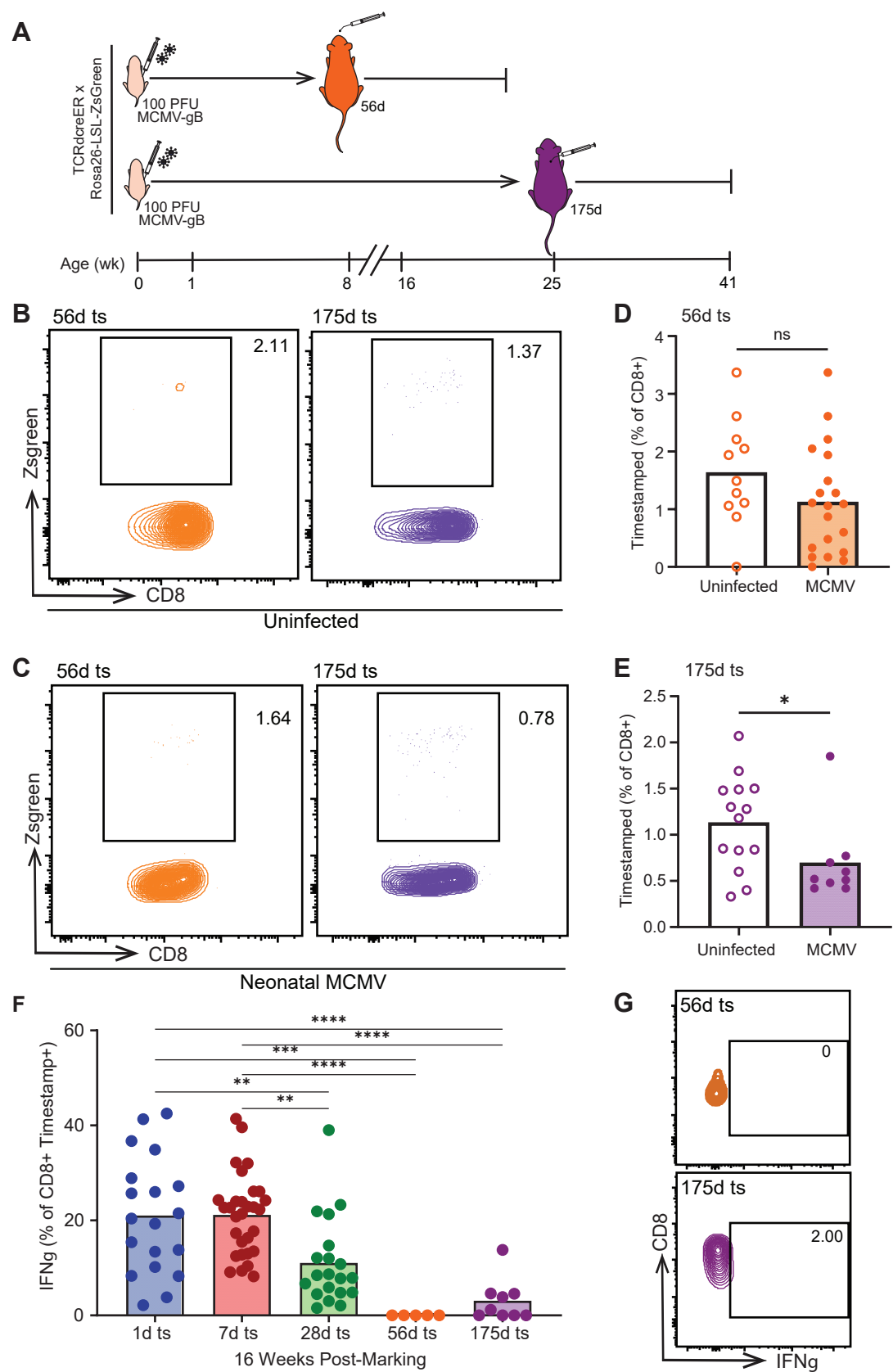

Supplement: S3 Fig — (A) Experimental schematic. Newborn TCRδcreERT2 x ZsGreen mice were infected with MCMV-gB at birth. Uninfected mice were injected with PBS as control. Mice were given tamoxifen at 56 or 175 days post-birth to ‘timestamp’ CD8 + T cells with a Zsgreen fluorescent tag. Mice were bled at 16 weeks post-marking. (B-C) representative contour plots of Zsgreen timestamping in the CD8 + populations of uninfected (B) or neonatally infected (C) mice. (D-E) Quantification of timestamped CD8 + T cells in 56 day (D) and 175 day (E) timestamp mice. (F-G) CD8 + T cells from the blood of 1d, 7d, 28d, 56d, and 175d marked mice at 16 weeks post-marking were gB peptide stimulated with BFA for 4 hours. Cells were then intracellularly stained for effector IFNγ. (F) Quantification of IFNγ production in indicated ‘timestamped’ groups. (G) Representative contour plots of IFNγ production in 56d (top) and 175d (bottom) timestamped cells. For D and E, Unpaired t-tests with Mann-Whitney test for correction were performed. Results are shown as mean only. For F) an one-way ANOVA with Tukey’s multiple comparisons test was performed. *p < 0.05 **p < 0.01, ****p < 0.0001. (PDF) [file ppat.1013382.s003.pdf]

**A**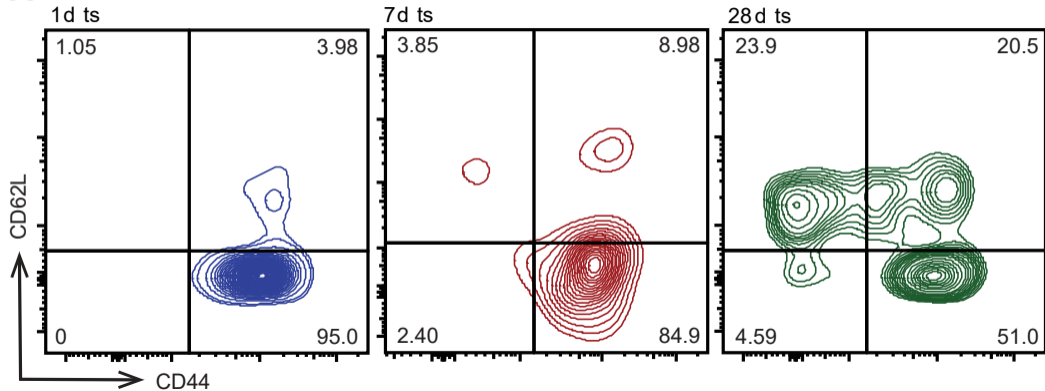**B**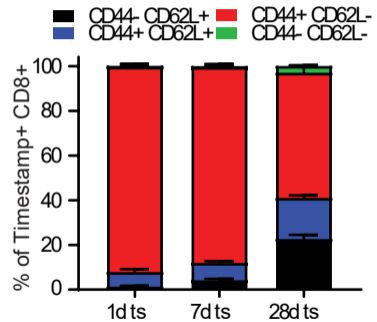

Supplement: S4 Fig — Newborn TCRδcreERT2 x ZsGreen mice were infected with MCMV-gB at birth. Uninfected mice were injected with PBS as control. Mice were given tamoxifen at 1 day, 7 days, or 28 days post-birth to ‘timestamp’ CD8 + T cells with a Zsgreen fluorescent tag. (A) Representative 2-way plot of CD44 vs CD62L on total timestamp CD8 + T cells. (B) Percentage of total timestamp CD8 + T cells that adopted an CD44 vs CD62L phenotype. For B, a two-way ANOVA with Tukey’s multiple comparisons test was performed. Results are shown as mean ± SEM. (PDF) [file ppat.1013382.s004.pdf]

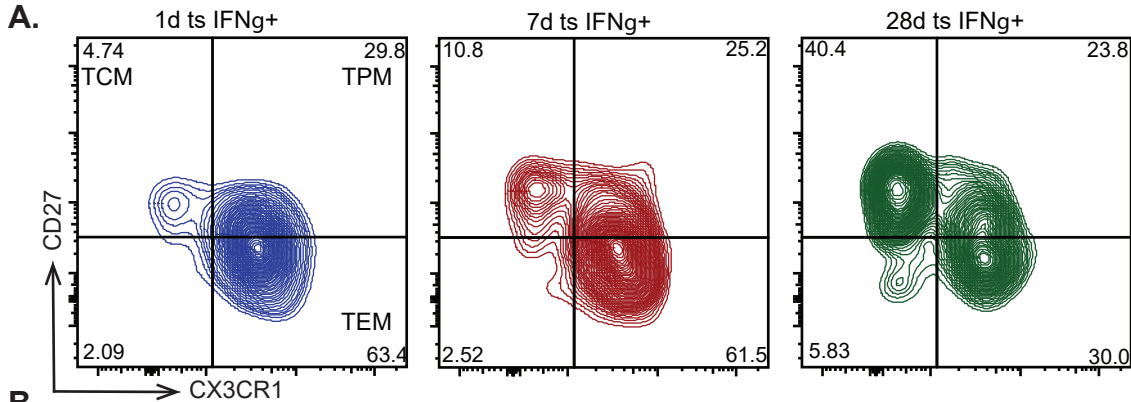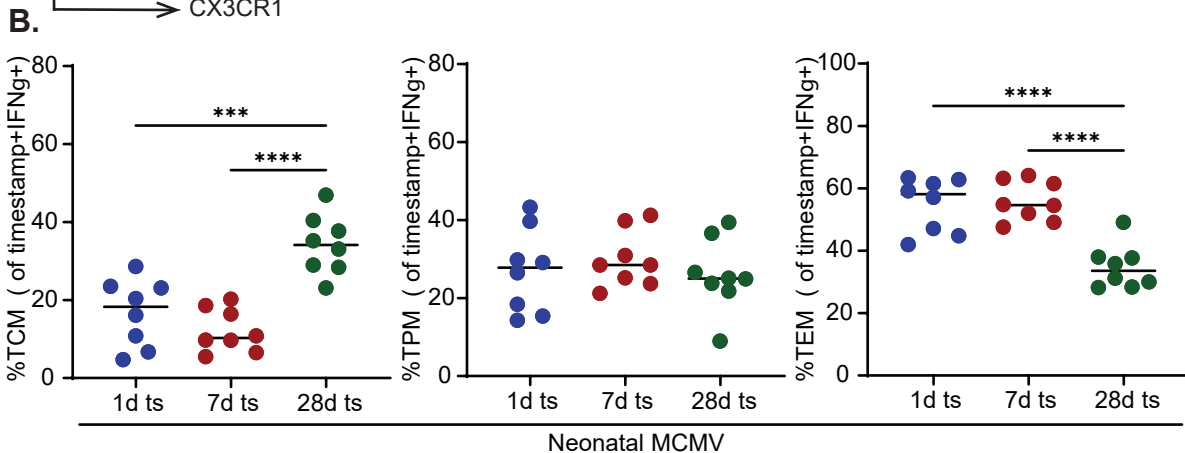

Supplement: S5 Fig — Newborn TCRδcreERT2 x ZsGreen mice were infected with MCMV-gB at birth. Uninfected mice were injected with PBS as control. Mice were given tamoxifen at 1 day, 7 days, or 28 days post-birth to ‘timestamp’ CD8 + T cells with a Zsgreen fluorescent tag. (A) Representative density plot of CD27 vs CX3CR1 within the IFNγ + sub gates where memory CD8 + T cells were identified as Central Memory (TCM, CD27 + CX3CR1-), Peripheral Memory (TPM, CD27 + CX3CR1+) or Effector Memory (TEM, CD27- CX3CR1+). (B) Quantification of TCM, TPM and TEM phenotype of 1d, 7d and 28d timestamped mice within the IFNγ+ population (N = 8 mice). For statistical test of more than two-groups, an ordinary One-way ANOVA with Tukey’s multiple comparisons test was performed. Results are shown as mean ± SD or mean only. ***p < 0.001, ****p < 0.0001. (PDF) [file ppat.1013382.s005.pdf]

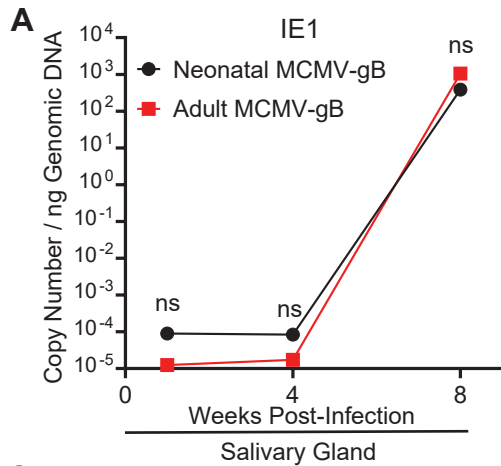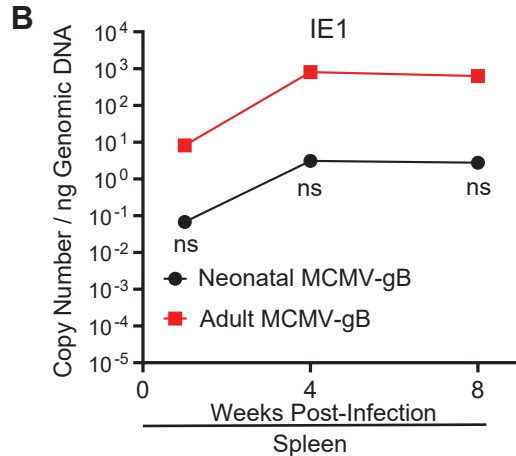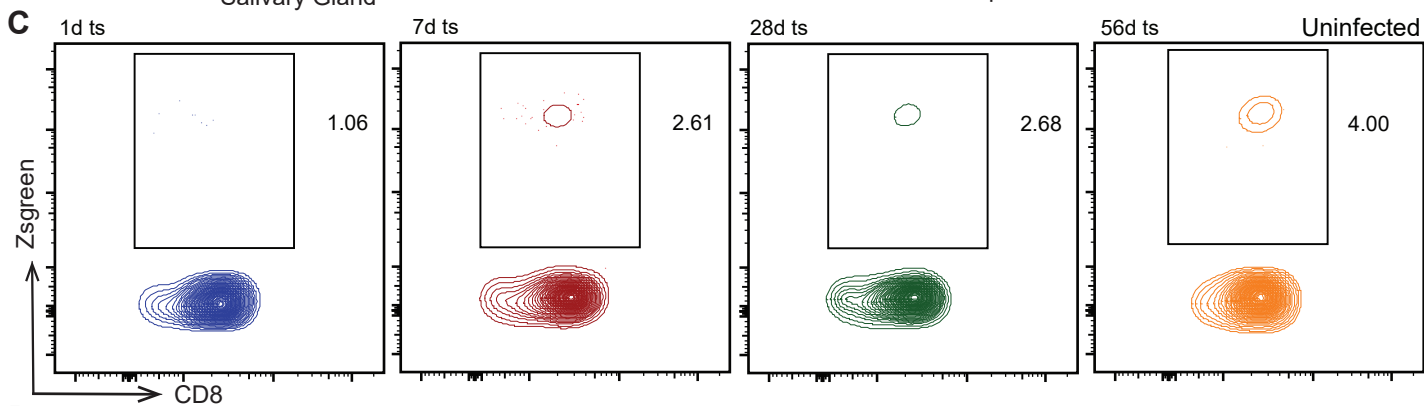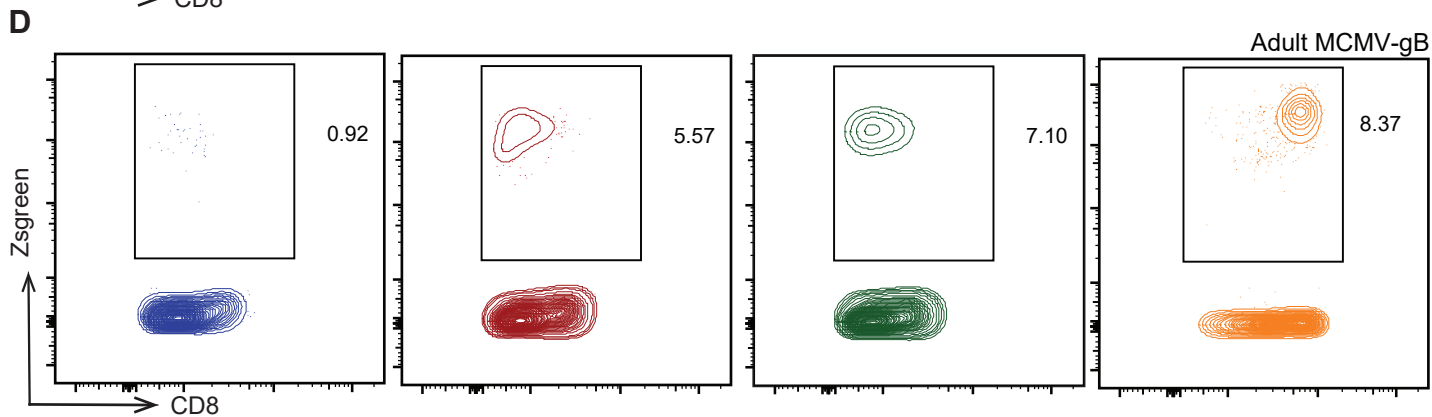

Supplement: S6 Fig — Quantification of viral replication in neonatal (black) or adult (red) MCMV-gB infection at indicated times post infection as measured by IE1 copy number within the (A) salivary gland and (B) spleen. (C-D) Adult mice were infected with MCMV-gB at 56 days post-birth. Uninfected mice were injected with PBS as control. Mice were given tamoxifen at 1 day, 7 days, 28 days or 56 days post-birth to ‘timestamp’ CD8 + T cells with a Zsgreen fluorescent tag. Mice were bled at 16 weeks post-birth and circulating Zsgreen+ CD8 + T cells were examined by flow cytometry. (C) Representative contour plots of uninfected 1d, 7d, 28d and 56d timestamp mice. (D) Representative contour plots of infected 1d, 7d, 28d and 56d timestamp mice. A two-way ANOVA with Bonferroni test correction was performed. (PDF) [file ppat.1013382.s006.pdf]

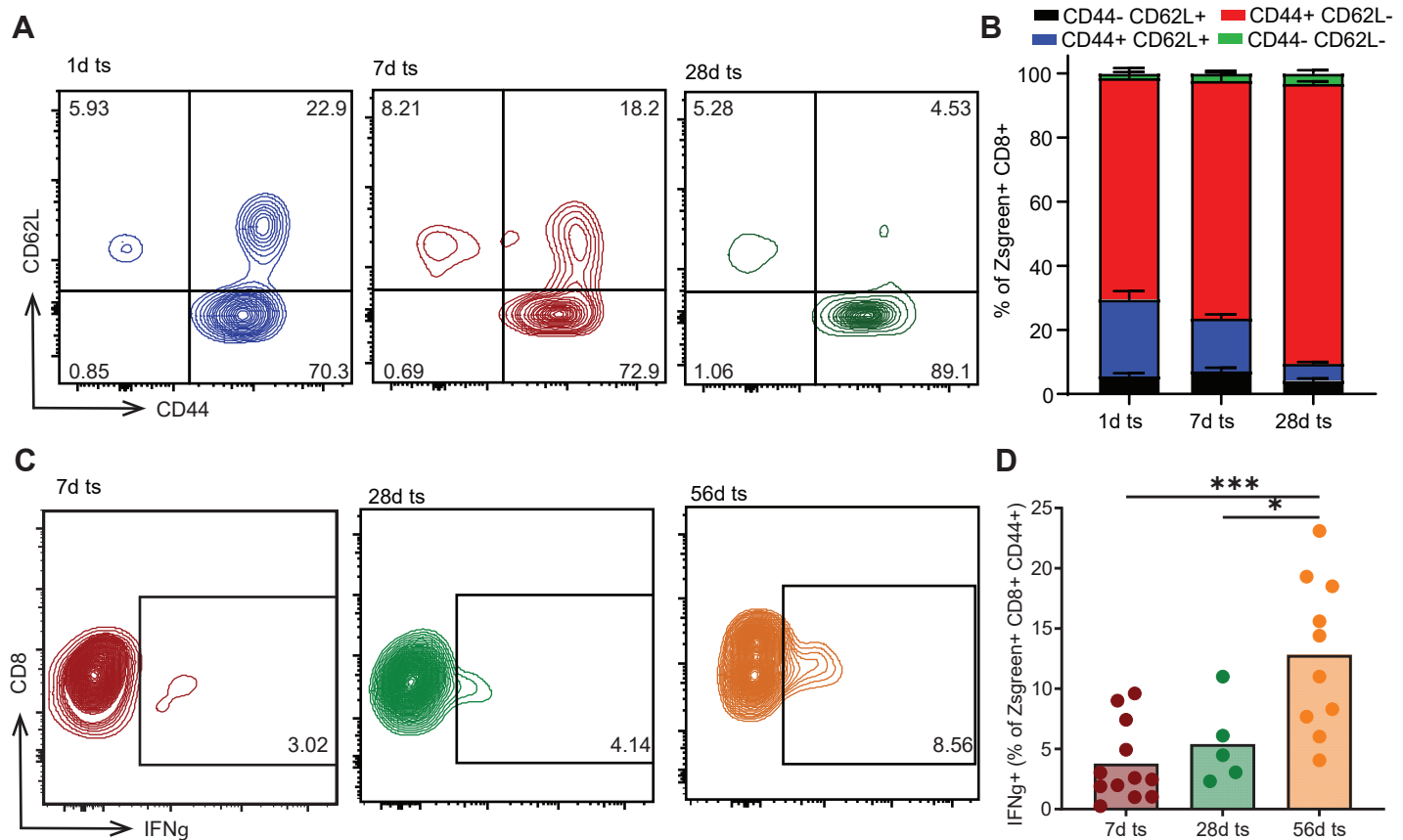

Supplement: S7 Fig — Adult timestamp mice were infected with MCMV-gB at 56 days post-birth and spleens were collected from adults at >24 weeks post-birth. CD8 + T cells were stained for CD44 vs CD62L to determine differentiation status. (A) Representative 2-way plot of CD44 vs CD62L on total timestamp CD8 + T cells. (B) Quantification of CD44 vs CD62L on total timestamp CD8 + T cells. (C-D. CD8 + T cells from the blood were given gB peptide stimulation with BFA was performed for 4 hours (N = 5–12 mice per group) (C) representative contour plots of and (D) statistical analysis of IFNγ production. For B) a two-way ANOVA with Bonferroni test correction was performed. For C) an ordinary One-way ANOVA with Tukey’s multiple comparisons test was performed. Results are shown as mean ± SEM. *p < 0.05, ***p < 0.001. (PDF) [file ppat.1013382.s007.pdf]

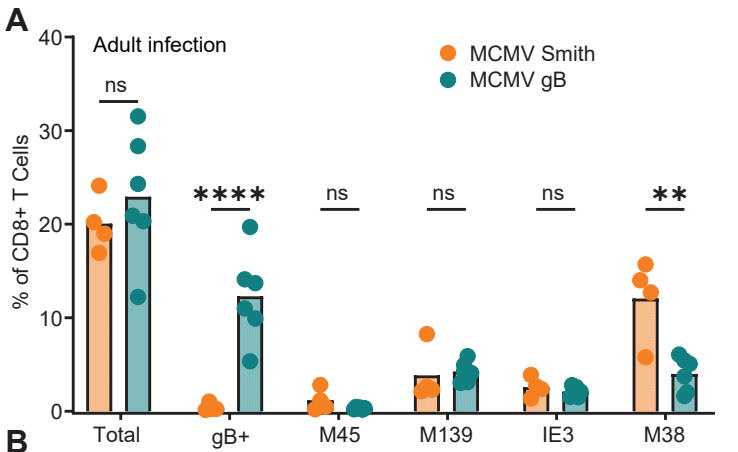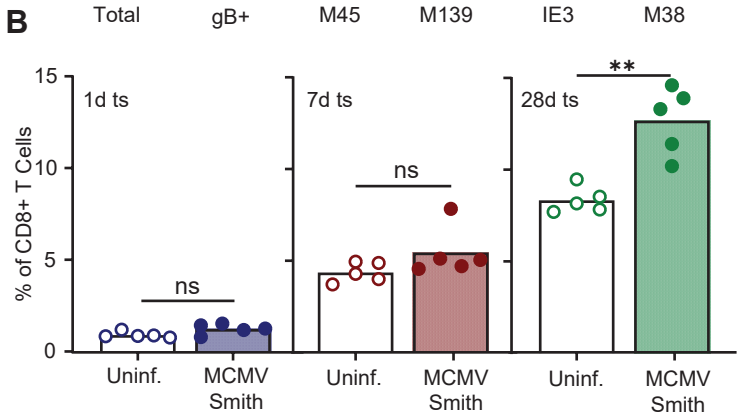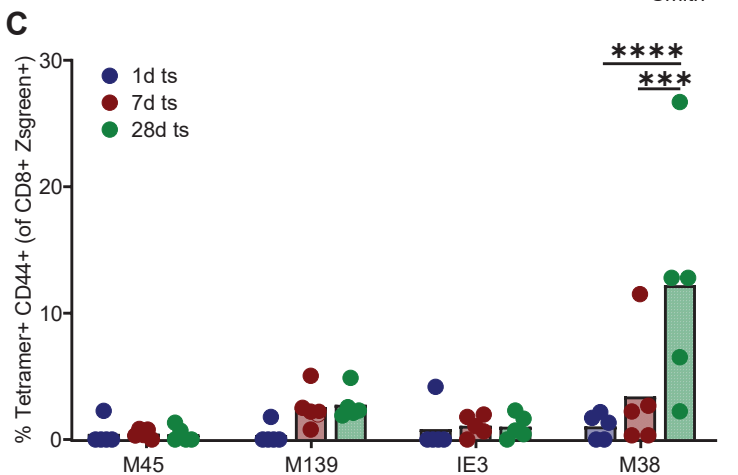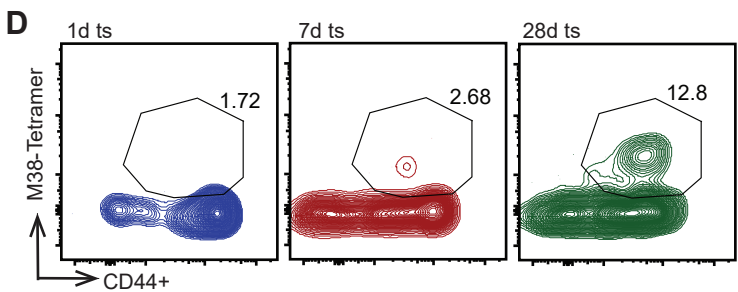

Supplement: S8 Fig — Adult mice were infected with MCMV-Smith or MCMV-gB (as indicated) at 56 days post-birth. (A) Proportion of responding CD8 + T cells pool to indicated T cell epitope, measured 6 wk following infection, N = 4–6. (B) Adult timestamp mice were infected with MCMV-Smith at 56 days post-birth. Uninfected mice were injected with PBS as control. Mice were given tamoxifen at 1 day, 7 days, 28 days or 56 days post-birth to ‘timestamp’ CD8 + T cells with a Zsgreen fluorescent tag. Mice were bled at 4 weeks post infection and ZsGreen+ percentage was measured (N = 5). (C) Percentage of epitope-specific, tetramer+ Zsgreen+ T cells at 4 weeks post infection (N = 5). (D) Representative contour plots showing M38-specific (inflating) ZsGreen+ CD8 + T cells at 4 weeks post infection. For A and C) a two-way ANOVA with Bonferroni test correction was performed. For B) an unpaired t-test with Mann-Whitney test for correction was performed. (PDF) [file ppat.1013382.s008.pdf]

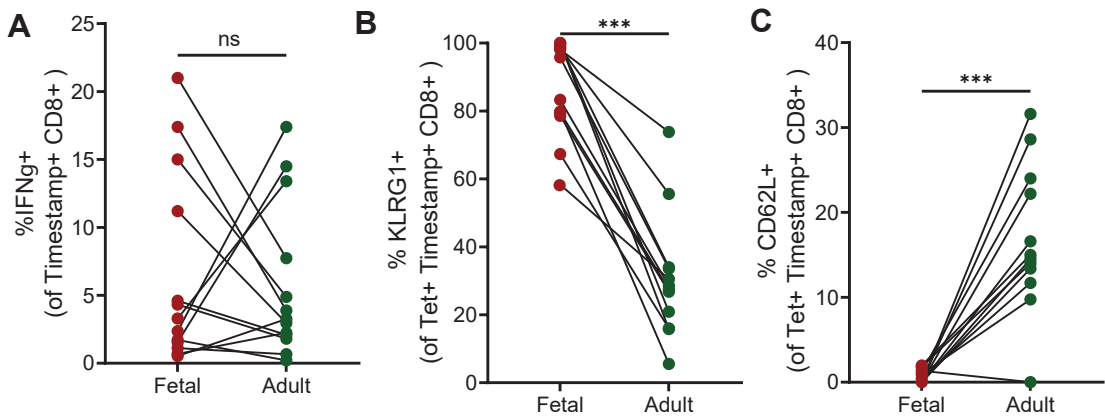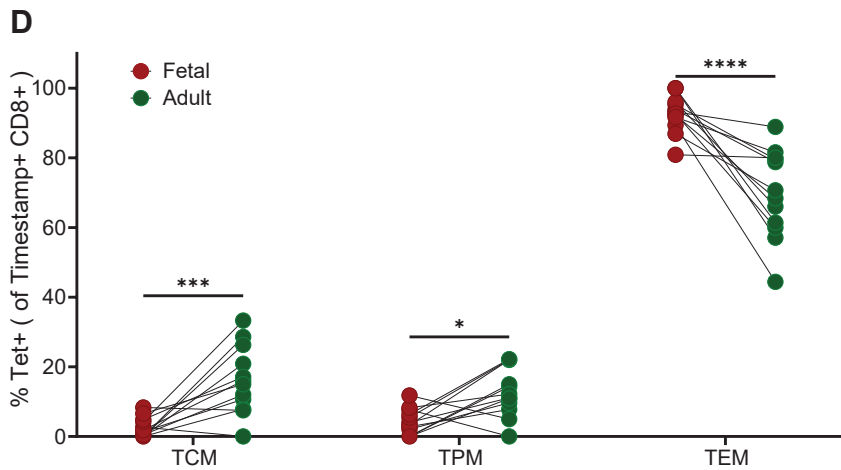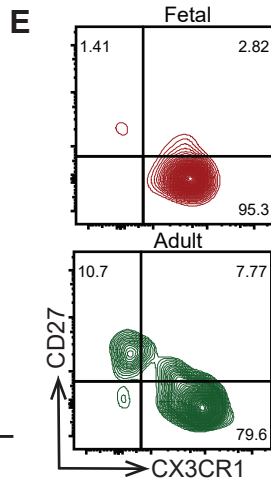

Supplement: S9 Fig — Newborn thymuses were collected from TdTomato+ timestamp reporter mice and surgically transplanted under the kidney capsule into adult (>8 week) Zsgreen+ timestamp reporter mice. Mice were administered tamoxifen for 3 days and then infected with MCMV-gB two days after marking. CD8 + T cell phenotypes were measured by flow cytometry at 16 week post infection. (A) Quantification of % of timestamped CD8 + T cells producing IFNγ. (B) Quantification of % of timestamped CD8 + T cells that express KLRG1. (C) Quantification of % of timestamped CD8 + T cells that express CD62L. (D) Quantification of memory CD8 + T cells identified as Central Memory (TCM, CD27 + CX3CR1-), Peripheral Memory (TPM, CD27 + CX3CR1+) or Effector Memory (TEM, CD27- CX3CR1+). (E) Representative contour plots showing previously indicated memory populations in fetal- (top) and adult-(bottom) derived RTEs. For A-C) an paired t-test with Wilcoxon matched pairs signed rank test performed for correction. For D) A two-way repeated measures ANOVA with Bonferroni test correction was performed. *p < 0.05, ***p < 0.001, ****p < 0.0001. (PDF) [file ppat.1013382.s009.pdf]
